# Psycho-social factors associated with mental resilience in the Corona lockdown

**DOI:** 10.1038/s41398-020-01150-4

**Published:** 2021-01-21

**Authors:** Ilya M. Veer, Antje Riepenhausen, Matthias Zerban, Carolin Wackerhagen, Lara M. C. Puhlmann, Haakon Engen, Göran Köber, Sophie A. Bögemann, Jeroen Weermeijer, Aleksandra Uściłko, Netali Mor, Marta A. Marciniak, Adrian Dahl Askelund, Abbas Al-Kamel, Sarah Ayash, Giulia Barsuola, Vaida Bartkute-Norkuniene, Simone Battaglia, Yaryna Bobko, Sven Bölte, Paolo Cardone, Edita Chvojková, Kaja Damnjanović, Joana De Calheiros Velozo, Lena de Thurah, Yacila I. Deza-Araujo, Annika Dimitrov, Kinga Farkas, Clémence Feller, Mary Gazea, Donya Gilan, Vedrana Gnjidić, Michal Hajduk, Anu P. Hiekkaranta, Live S. Hofgaard, Laura Ilen, Zuzana Kasanova, Mohsen Khanpour, Bobo Hi Po Lau, Dionne B. Lenferink, Thomas B. Lindhardt, Dávid Á. Magas, Julian Mituniewicz, Laura Moreno-López, Sofiia Muzychka, Maria Ntafouli, Aet O’Leary, Ilenia Paparella, Nele Põldver, Aki Rintala, Natalia Robak, Anna M. Rosická, Espen Røysamb, Siavash Sadeghi, Maude Schneider, Roma Siugzdaite, Mirta Stantić, Ana Teixeira, Ana Todorovic, Wendy W. N. Wan, Rolf van Dick, Klaus Lieb, Birgit Kleim, Erno J. Hermans, Dorota Kobylińska, Talma Hendler, Harald Binder, Inez Myin-Germeys, Judith M. C. van Leeuwen, Oliver Tüscher, Kenneth S. L. Yuen, Henrik Walter, Raffael Kalisch

**Affiliations:** 1 Research Division of Mind and Brain, Department of Psychiatry and Psychotherapy CCM, Charité–Universitätsmedizin Berlin, Corporate Member of Freie Universität Berlin, Humboldt-Universität zu Berlin, and Berlin Institute of Health, Berlin, Germany; 2 Faculty of Philosophy, Berlin School of Mind and Brain, Humboldt-Universität zu Berlin, Berlin, Germany; 3 Neuroimaging Center (NIC), Focus Program Translational Neuroscience (FTN), Johannes Gutenberg University Medical Center, Mainz, Germany; 4 Leibniz Institute for Resilience Research (LIR), Mainz, Germany; 5 Research Group Social Stress and Family Health, Max Planck Institute for Cognitive and Brain Sciences, Leipzig, Germany; 6 Department of Psychology, University of Oslo, Oslo, Norway; 7 Faculty of Medicine and Medical Center, Institute of Medical Biometry and Statistics, University of Freiburg, Freiburg, Germany; 8 Freiburg Center for Data Analysis and Modelling, University of Freiburg, Freiburg, Germany; 9 Donders Institute for Brain, Cognition, and Behaviour, Radboud University Medical Center, Nijmegen, The Netherlands; 10 Center for Contextual Psychiatry, Department of Neurosciences, KU Leuven, Leuven, Belgium; 11 Faculty of Psychology, University of Warsaw, Warsaw, Poland; 12 Tel Aviv Sourasky Medical Center, Sagol Brain Institute Tel Aviv, Tel Aviv, Israel; 13 Faculty of Medicine, Tel Aviv University, Tel Aviv, Israel; 14 Division of Experimental Psychopathology and Psychotherapy, Department of Psychology, University of Zurich, Zurich, Switzerland; 15 Department of Psychiatry, Psychotherapy and Psychosomatics, Psychiatric University Hospital (PUK), University of Zurich, Zurich, Switzerland; 16 University of Bergamo, Bergamo, Italy; 17 MRC Cognition and Brain Sciences Unit, University of Cambridge, Cambridge, UK; 18 Faculty of Business and Technologies at Utena University of Applied Sciences, Utena, Lithuania; 19 Center for Studies and Research in Cognitive Neuroscience, Department of Psychology, University of Bologna, Bologna, Italy; 20 Faculty of Psychology, University of Economics and Human Sciences, Warsaw, Warsaw, Poland; 21 Center for Psychiatry Research, Department of Women’s and Children’s Health, Karolinska Institutet Center of Neurodevelopmental Disorders (KIND), Karolinska Institutet and Stockholm Health Care Services, Region Stockholm, Stockholm, Sweden; 22 Child and Adolescent Psychiatry, Stockholm Health Care Services, Region Stockholm, Stockholm, Sweden; 23 Curtin Autism Research Group, School of Occupational Therapy, Social Work and Speech Pathology, Curtin University, Perth, WA Australia; 24 Department of Psychological Methods, University of Amsterdam, Amsterdam, The Netherlands; 25 Laboratory for Experimental Psychology, Department of Psychology, University of Belgrade, Belgrade, Serbia; 26 Swiss Center for Affective Sciences, University of Geneva, Geneva, Switzerland; 27 Laboratory for Behavioral Neurology and Imaging of Cognition, Department of Neuroscience, Medical School, University of Geneva, Geneva, Switzerland; 28 Department of Psychiatry and Psychotherapy, Semmelweis University, Budapest, Hungary; 29 Department of Cognitive Science, Budapest University of Technology and Economics, Budapest, Hungary; 30 Clinical Psychology Unit for Intellectual and Developmental Disabilities, Faculty of Psychology and Educational Sciences, University of Geneva, Geneva, Switzerland; 31 Concentris Research Management GmbH, Fürstenfeldbruck, Germany; 32 Department of Psychiatry and Psychotherapy, Johannes Gutenberg University Medical Center, Mainz, Germany; 33 Faculty of Humanities and Social Sciences, University of Zagreb, Zagreb, Croatia; 34 Department of Psychology, Faculty of Arts, Comenius University in Bratislava, Bratislava, Slovak Republic; 35 Center for Psychiatric Disorders Research, University in Bratislava, Science Park Comenius, Bratislava, Slovak Republic; 36 Department of Psychiatry, Faculty of Medicine, Comenius University in Bratislava, Bratislava, Slovak Republic; 37 PROMENTA Research Centre, Department of Psychology, University of Oslo, Oslo, Norway; 38 Leuven Research and Development, Spin-off and Innovation Unit, KU Leuven, Leuven, Belgium; 39 University of Tehran, Tehran, Iran; 40 Department of Counselling and Psychology, Hong Kong Shue Yan University, Hong Kong, Hong Kong; 41 Center of Functionally Integrative Neuroscience (CFIN) and MINDLab, Department of Clinical Medicine, Århus University, Århus, Denmark; 42 Department of Psychiatry, University of Cambridge, Cambridge, UK; 43 Sleep Research Unit, First Department of Psychiatry, National and Kapodistrian University of Athens, Athens, Greece; 44 Department of Psychiatry, Psychosomatics and Psychotherapy, University Hospital Frankfurt, Frankfurt am Main, Germany; 45 Institute of Psychology, University of Tartu, Tartu, Estonia; 46 Institut des Sciences Cognitives Marc Jeannerod, Lyon, France; 47 Faculty of Social Services and Health Care, LAB University of Applied Sciences, Lahti, Finland; 48 College of Inter-faculty Individual Studies in Mathematics and Natural Sciences, University of Warsaw, Warsaw, Poland; 49 Faculty of Social Studies, Department of Psychology, Masaryk University, Brno, Czech Republic; 50 Johannes Gutenberg University Mainz, Mainz, Germany; 51 Department of Experimental Clinical and Health Psychology, Ghent University, Gent, Belgium; 52 Department of Experimental Psychology, University of Oxford, Oxford, UK; 53 Department of International Business, Tunghai University, Taichung City, Taiwan; 54 Institute of Psychology, Goethe University Frankfurt, Frankfurt am Main, Germany; 55 School of Psychological Science, Tel Aviv University, Tel Aviv, Israel; 56 Sagol School of Neuroscience, Tel Aviv University, Tel Aviv, Israel

**Keywords:** Human behaviour, Diagnostic markers

## Abstract

The SARS-CoV-2 pandemic is not only a threat to physical health but is also having severe impacts on mental health. Although increases in stress-related symptomatology and other adverse psycho-social outcomes, as well as their most important risk factors have been described, hardly anything is known about potential protective factors. Resilience refers to the maintenance of mental health despite adversity. To gain mechanistic insights about the relationship between described psycho-social resilience factors and resilience specifically in the current crisis, we assessed resilience factors, exposure to Corona crisis-specific and general stressors, as well as internalizing symptoms in a cross-sectional online survey conducted in 24 languages during the most intense phase of the lockdown in Europe (22 March to 19 April) in a convenience sample of *N* = 15,970 adults. Resilience, as an outcome, was conceptualized as good mental health despite stressor exposure and measured as the inverse residual between actual and predicted symptom total score. Preregistered hypotheses (osf.io/r6btn) were tested with multiple regression models and mediation analyses. Results confirmed our primary hypothesis that positive appraisal style (PAS) is positively associated with resilience (*p* < 0.0001). The resilience factor PAS also partly mediated the positive association between perceived social support and resilience, and its association with resilience was in turn partly mediated by the ability to easily recover from stress (both *p* < 0.0001). In comparison with other resilience factors, good stress response recovery and positive appraisal specifically of the consequences of the Corona crisis were the strongest factors. Preregistered exploratory subgroup analyses (osf.io/thka9) showed that all tested resilience factors generalize across major socio-demographic categories. This research identifies modifiable protective factors that can be targeted by public mental health efforts in this and in future pandemics.

## Introduction

Pandemics can induce high levels of stress and result in mental health problems, including depression, anxiety, and posttraumatic stress disorder symptomatology^1–3^. Marked effects have also been reported during the current severe acute respiratory syndrome coronavirus 2 (SARS-CoV-2) pandemic in Asia, Europe, and North America^4–6^. Measures of social distancing and quarantine aimed to curtail the spread of the pathogen can have additional detrimental psychological effects^7^, as has also been seen during the current pandemic^8–10^. First evidence from Italy and the United States also indicates other psycho-social consequences, in particular increased loneliness and domestic violence^11,12^. On this basis, urgent calls for mental health science in the SARS-CoV-2 pandemic have been issued and it has also been pointed out that such research is important to better prepare individuals and health systems for future pandemics or potential other waves of the current one (e.g., see refs. ^13,14^).

We here present data collected through a globally available online survey between 22 March and 19 April (23:59 h). This timeframe corresponds to a phase of the pandemic when severe lockdown measures were in place in many of the most affected European countries and where the stresses related to the physical threat posed by the virus and the uncertainty about the further course of the pandemic mixed with the specific challenges posed by the curfews and the other movement and contact restrictions. The undesirable side effects of lockdown include social isolation and economic restrictions but also that professional psychological or psychiatric help is even more difficult to obtain than in normal times^15,16^. Although several studies have already identified factors that increase the risk for developing stress-related symptoms or disorders in the SARS-CoV-2 pandemic, and specifically during lockdown situations (e.g., see refs. ^5,8–10,17–22^), little is known about factors that shield against such effects. First reports highlight negative associations between mental health problems and various forms of social support^17,23,24^, financial security^25^, availability of information^26^, and self-efficacy^24^.

Our study uses a resilience framework, founded on a definition of resilience as maintenance or quick recovery of mental health during and after times of adversity^27–29^. In this perspective, resilience is an outcome consisting in good mental health despite stressor exposure, and its operationalization and quantification necessarily involves an assessment of the stressors individuals are confronted with ref. ^27^. On this basis, one can then try to identify the social, psychological, and biological factors associated with that outcome. Provided individual differences in stressor exposure are appropriately accounted for, an observed positive association of any factor with mental health can then be interpreted as expressing a protective function of that factor against the mental health effects of the assessed stressors.

Most knowledge about resilience factors stem from individual-level traumata or challenges, or from commonly experienced catastrophes such as natural disasters or terror attacks^27,29,30^, but there are no systematic studies specifically investigating resilience factors effective in pandemics^7^. Such knowledge, however, is crucial for developing mental health protection measures suitable for pandemic-like situations.

In our global internet-based cross-sectional survey (DynaCORE-C—the DynaMORE cross-sectional survey study on psychological resilience to the mental health consequences of the Corona crisis; by the EU project DynaMORE, www.dynamore-project.eu), we assessed potential psycho-social resilience factors and their association with outcome-based resilience. Our primary hypothesis was that positive appraisal style (PAS) is a resilience factor. PAS is a new construct developed based on positive appraisal style theory of RES (PASTOR^31^) and predictive of outcome-based resilience in own, yet unpublished prospective-longitudinal studies (Supplement Section 2.2.2). At survey start on 22 March, measures of quarantine, social distancing, or curfew were already in place in many European countries. A first interim analysis in 5000 European respondents covered the time until 1 April (preregistration: osf.io/r6btn^32^). This second interim analysis in *N* = 15,790 participants extends until 20 April, when first restrictions began to be eased in some European countries highly represented in our sample. Thereby, we cover the most intense phase of the lockdown in Europe. Further, the analysis now also includes data sets from non-European countries and we use the considerably larger sample size to also conduct exploratory subgroup analyses aiming at establishing to what extent findings about resilience factors generalize across socio-demographic groups (gender, age, country of residence, household income, years of education, past or present mental health diagnosis) and whether these groups differ in their effect sizes (for preregistration, see osf.io/thka9). If effects generalize across subgroups, i.e., if a resilience factor has positive effects at all levels of a socio-demographic covariate, the identified resilience factor is likely valid in the broader population from which the current sample was drawn. If there are pronounced subgroup differences, this would yield valuable information on which resilience factors might be the most promising targets of preventive interventions in different subgroups.

## Participants and methods

### Sample

Participants (all genders, older than 18 years, no other in- or exclusion criteria) were recruited using a snowball sampling approach started on 22 March 2020 via the social media, mailing lists, and a general media campaign. Data collection via the SurveyMonkey platform (www.surveymonkey.de) was anonymous. Participants gave informed consent electronically. The study was approved by the ethics committee of the State Medical Board of Rhineland-Palatinate, Germany, and was conducted in accordance with the Declaration of Helsinki.

There was no initially planned sample size and the survey was paused on 8 July 2020, when recruitment had been low for several weeks. In the beginning, the survey questionnaire was available in English and German only; by 1 April, ten further languages had been added, and by 20 April, a total of 24 languages were available (Supplement 1).

By 20 April, 26,348 survey data sets were registered. Data cleaning (see below) removed 7653 data sets. Of the remaining 18,695 valid data sets, 2905 (15.5%) were incomplete (for characteristics, see Supplement 4). The vast majority of incomplete data sets did not contain complete answers to the stressor exposure questions at the end of the questionnaire, which are however needed to calculate the resilience outcome score (see below). Therefore, this analysis focuses on the 15,790 valid participants providing complete data. (It is noteworthy that there is a discrepancy with the preregistration of the second interim analysis at osf.io/thka9, which mentions 14,460 complete data sets. This latter number arises from an erroneous calculation that only considered data sets complete when the optional free-text questions had also been answered.) For characteristics of the final sample, see Supplementary Table S1. The sample contains a large proportion of women, younger people, people with higher education, Europeans, students, and employees working in research and/or education, as well as in health care.

### Independent variables (resilience factors) and covariates

Information on the survey questionnaire, derived variables and indices, and socio-demographic and health covariates are given in Supplement 1, and Supplementary Tables S2 and S3. Shortly after the start of the survey, a question on past or present diagnosed mental health conditions was added. Among the *n* = 15,384 analyzed participants interrogated about a diagnosis, 22.9% affirmed the question (Supplementary Table S4). Independent variables for which we hypothesized directed associations with resilience (psycho-social resilience factors) based on existing literature^27,29,30^ and own ongoing, unpublished work are as follows: PAS (primary hypothesis), perceived social support, a perceived increase in social support during the Corona crisis, optimism, perceived general self-efficacy, perceived good stress, neuroticism (inverse), behavioral coping style, and positive appraisal specifically of the Corona crisis (key secondary hypotheses). We also pre-formulated two mediation hypotheses. For details, see Supplement 2.2 and Supplementary Table S5. For the development of the PAS instrument specifically, see Supplement 2.2.2.

### Assessment of stressors

The questionnaire includes a detailed assessment of stressors participants have been exposed to in the past 2 weeks. As mentioned, measurement of stressor exposure is an important, although often neglected ingredient of resilience research, because resilience is only a meaningful concept when adversity is present (see below and refs. ^27,28^). We differentiate between exposure to general stressors (*E*_G_), as they may also occur in normal times but may well have been exacerbated by the Corona crisis (11 broad classes of stressors such as family conflicts, physical health problems, or financial problems), as well as exposure to stressors specific for the Corona crisis (*E*_S_) (29 items such as COVID-19 symptoms, belonging to a risk group for serious COVID-19 symptoms, loss of social contact, or problems arranging childcare). We quantified *E*_G_ and *E*_S_ by the sum count of the reported stressors, weighted by their rated severity, and also combine both stressor categories into a common index *E*_C_ by averaging the *z*-normalized *E*_G_ and *E*_S_ sum counts. See Supplement 2.3.2 and Supplementary Table S6.

### Measurement of resilience (dependent variable)

In keeping with the current conceptualizations of individual (mental or psychological) resilience^27–29^, we define resilience as an outcome of good mental health despite exposure to adversity. We measured outcome-based resilience by relating self-reported changes in mental health problems *P* over the past 2 weeks (internalizing symptoms assessed with the General Health Questionnaire GHQ-12^33^) to the self-reported stressor exposure *E* during the same time window^31^. The *E*–*P* regression curve can be considered the normative predicted reactivity of mental health to stressor exposure in the sample. An individual’s *P* score lying above the curve then expresses relative over-reactivity, a score lying below the curve relative under-reactivity. Following refs. ^34,35^, we therefore used individuals’ inverse residuals onto the regression curve as a measure of their resilience, RES^36^. This normative modeling method has the advantage that it inherently corrects for individual differences in stressor exposure (see also Supplement 2.3.4). We differentiate between resilience to all stressors combined (RES_C_), to general stressors (RES_G_), and to Corona crisis-specific stressors (RES_S_).

### Data cleaning

Data cleaning removed 7653 participants who gave invalid age responses, indicated that they were underage, terminated the survey before providing responses to the initial socio-demographic questions, provided their household income on a prefinal scale, which was adapted in the early phase of data collection (see Supplement 1), or failed to complete follow-up questions on socio-demographic characteristics.

### Statistical analyses

The statistical methods used in this second interim analysis (preregistration: osf.io/thka9) are identical to the first interim analysis (preregistration: osf.io/r6btn^32^) and were complemented by additional exploratory subgroup analyses. For the main analyses, we first assessed the influence of the socio-demographic and health covariates on RES using separate univariate regression analyses and included all covariates surviving a likelihood ratio test at *p* < 0.2 in all further analyses (Supplementary Table S3). The directed hypotheses about resilience factors (above and Supplementary Table S5) were tested separately using multiple regressions. The two planned mediation analyses (Supplementary Table S5) were conducted following a Baron and Kenny approach. The preregistration states that significance of the indirect paths in the mediation analyses will be determined with the distribution-of-the-product method. Due to convergence issues with that method for inferring confidence intervals of the mediation effects, we switched to the asymptotic normal distribution method^37^. The α-level for all analyses was *p* < 0.01, two-tailed. These analyses were repeated for the subsample that had been interrogated about potential mental health conditions, adding past or present diagnosis as covariate. All results remained (data not shown).

We also considered partial correlations of independent variables (Supplementary Table S7). To identify the strongest resilience factors among the independent variables, we combined the variables and the included covariates in a LASSO (Least Absolute Shrinkage and Selection Operator) analysis, where the L_1_-Norm of the coefficients was penalized with a parameter *λ*^38^. The LASSO is used specifically as a sensitivity analysis for selecting important variables in multi-variable settings. We picked *λ* based on cross-validation to identify a subset of variables that is particularly suited for predicting RES. Optimal *λ* was defined as the *λ* that minimizes cross-validation error +1 SE, a criterion designed to select the simplest model whose accuracy is comparable to the best model^39,40^, thus minimizing risk of overfitting/maximizing generalizability.

To additionally explore subgroup effects, we used separate multiple regression analyses of each of the nine primary and key secondary hypotheses about resilience factors (see “Independent variables (resilience factors) and covariates”) where we added the interaction term between the main independent variable (resilience factor) and a socio-demographic covariate of interest. This was done separately for each covariate. Tested covariates were as follows: gender (subgroups: male, female), age (18–30, 31–45, 46–60, 61, or more years), country of residence (grouped as countries with 500 or more respondents, namely Belgium, Germany, Hong Kong, Hungary, Italy, the Netherlands, Poland, Serbia, or other), household income (0–4999, 5000–9999, 10,000–14,999, 15,000–24,999, 25,000–49,999, 50,000–74,999, 75,000–99,999, 100,000, or more €), years of education (<13, 13–16, 17–20, 21, or more years), and past or present mental health diagnosis (yes, no). The guidelines for the subgrouping of these covariates were as follows: to obtain approximately equally sized subgroups, to limit the number of subgroups per covariate as much as possible, while keeping sufficient resolution for meaningful interpretation, to obtain subgroups with sufficient size for reliable analysis, and to take into consideration obvious or theory-based subgroup boundaries (such as, e.g., male/female/diverse gender, mental health diagnosis, or not). To this end, the covariate data from the first interim analysis as well as the data about the distribution of gender, age, and countries of residence available on 19 April 2020 were taken into consideration (see Section 6.1 of the “Preregistration of the first interim analysis” in osf.io/r6btn). The gender category “diverse” was not included in the subgroup analyses because of its small size (*n* = 125) as was the mental health diagnosis category “not assessed” (in an early study phase). In these cases, the sample for the given analysis was reduced accordingly. We then explored the pattern of effect estimates that correspond to the main hypotheses for each level of the covariate, to qualitatively describe the influence of the covariate. For each level of a covariate, we also conducted combined regularized regression analysis (LASSO), to examine the relative strengths of resilience factors per subgroup.

Data cleaning and analysis were performed in R (v3.6.3, www.r-project.org/).

## Results

### Stressor exposure

The most frequently experienced general stressors (*E*_G_) were negative political events (reported by 83% of participants), followed by conflicts/disagreements in family, social, or professional settings (62%) and burdensome experiences at work/school/university or another occupation (61%). The general stressors experienced as most burdensome were death of a loved one (average severity rating 3.85, possible answer range 1–5), followed by separation from a loved one (3.56), and oneself or a close person experiencing mental health problems (3.29). The most frequently experienced Corona crisis-specific stressors (*E*_S_) were Corona-related media coverage (93%), closely followed by not being able to perform leisure activities (90%), loss of social contact (88%), and (feeling) restricted to leave home (86%). Most burdensome were the inability to attend a funeral of a family member/friend/loved one (3.75), family/friends/loved ones being at hospital, while one is restricted to visit them (3.66), and family/friends/loved ones being at increased risk for a serious course of the disease in case of an infection (3.5). See Supplementary Table S6 for details.

As the general stressor list contained items that might be exacerbated by the Corona crisis (such as negative political events or family conflicts), answers to this list might also be influenced by the crisis. In another sample providing detailed general stressor reports before the crisis, we have observed a qualitatively different pattern of experienced stressors (Supplement 2.3.2). In the current sample, we further found a high correlation between the general and the Corona crisis-specific stressor exposure scores *E*_G_ and *E*_S_, respectively (*R* = 0.66). Together, this indicates that the Corona crisis was the dominant source of stressors in the current sample. As the combined stressor exposure score *E*_C_ also explained most variance in mental health problems *P* (see Supplement 2.3.2), we focus on resilience to all stressors combined (RES_C_).

An effect of the crisis on participants’ mental health was suggested by high average *P* scores of 15.5 ± 6.2 (SD) (possible range 0–36; comp. 9.7 ± 4.9^41^ and 8.3^42^ in available representative samples from Europe). Participants with a past or present mental health condition had a higher average score (17.7 ± 6.9) than those without (14.9 ± 5.8; *t*(5036) = 21.86, *p* < 0.0001).

### Resilience factors

**Fig. 1:**
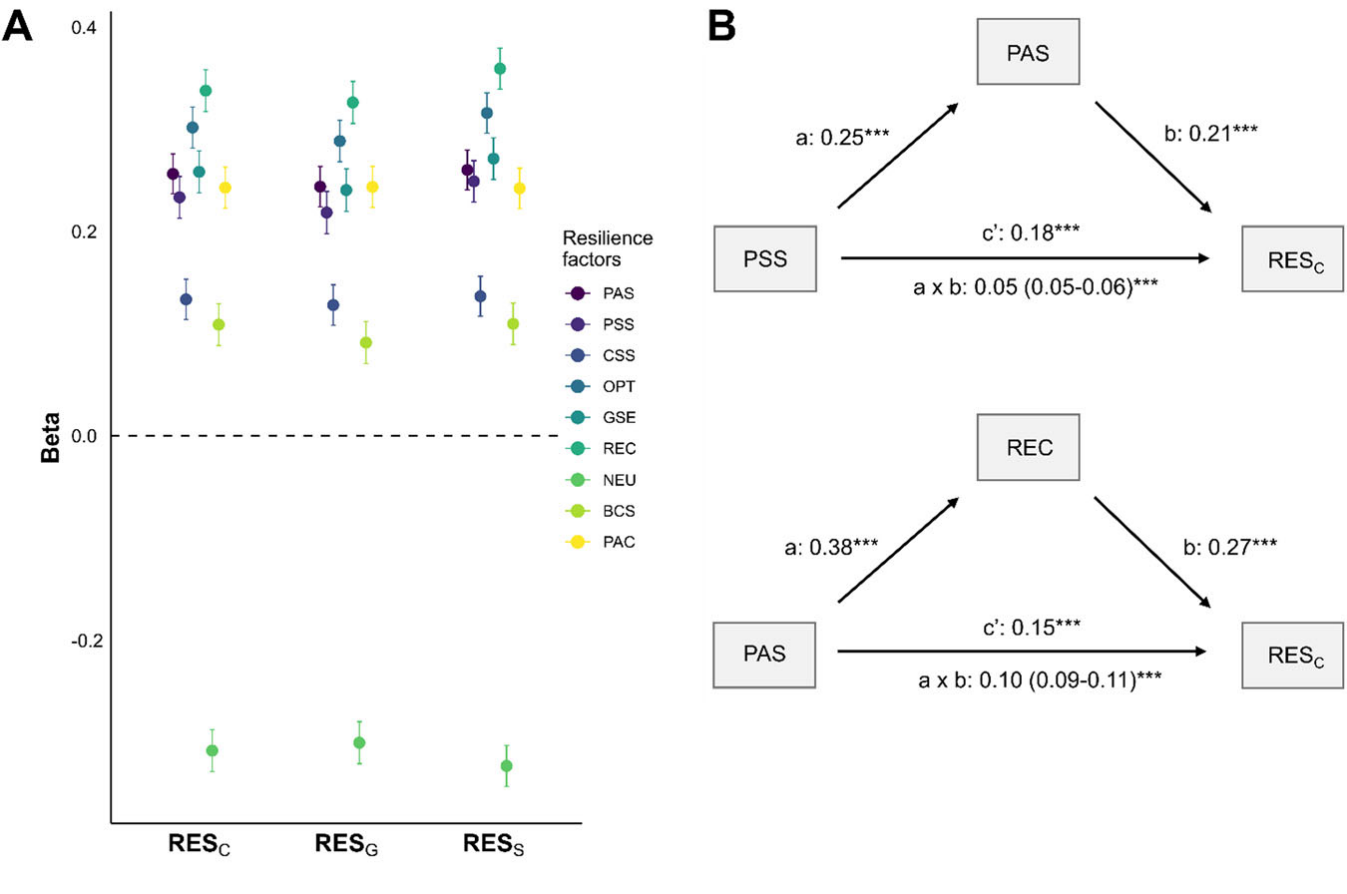
Associations of hypothesized resilience factors with outcome-based resilience (RES) and mediation effects. **A** Multiple regressions with covariates of resilience factors on resilience, calculated separately for each factor. Shown are the regression coefficients (*β*) and 99% confidence intervals (CI). Effects are similar for resilience to all stressors combined (RES_C_), resilience to general (RES_G_), and resilience to Corona-specific stressors (RES_S_). Resilience factors: PAS, positive appraisal style; PSS, perceived social support; CSS, perceived increase in social support during the Corona crisis; OPT, optimism; GSE, perceived general self-efficacy; REC, perceived good stress recovery; NEU, neuroticism; BCS, behavioral coping style; PAC, positive appraisal specifically of the Corona crisis. **B** Mediation analyses testing if the positive association of PSS with RES_C_ is mediated by PAS (top) and if the positive association of PAS on RES_C_ is mediated by REC (bottom). Shown are *β* of all paths. Indirect path *a* × *b*: *β* with 99% CI. ***p < 0.0001.

Our primary hypothesis was that RES_C_ is positively associated with PAS. Controlling for covariates, PAS explained significant additional variance in RES_C_ (adjusted *R*^2^ increase: 0.06, *p* < 0.0001). See Fig. 1A and Supplementary Tables S8–S10.

In agreement with the multifactorial nature of resilience^27,29,30^ all our key secondary hypotheses about resilience factors (see “Methods”) were also confirmed (all *p* < 0.0001, comp. Bonferroni threshold for multiple comparisons: *p* = 0.01/9 = 0.0011; Fig. 1A and Supplementary Tables S8–S10). Expectedly^43^, neuroticism had a strong negative influence.

We also predicted that the expected positive association of perceived social support with RES_C_ is positively mediated by its association with PAS, and that the expected positive association of PAS with RES_C_ is positively mediated by its association with perceived good stress response recovery (REC) (see Supplement 2.2.3). These hypotheses were also confirmed (Fig. 1B).

**Fig. 2:**
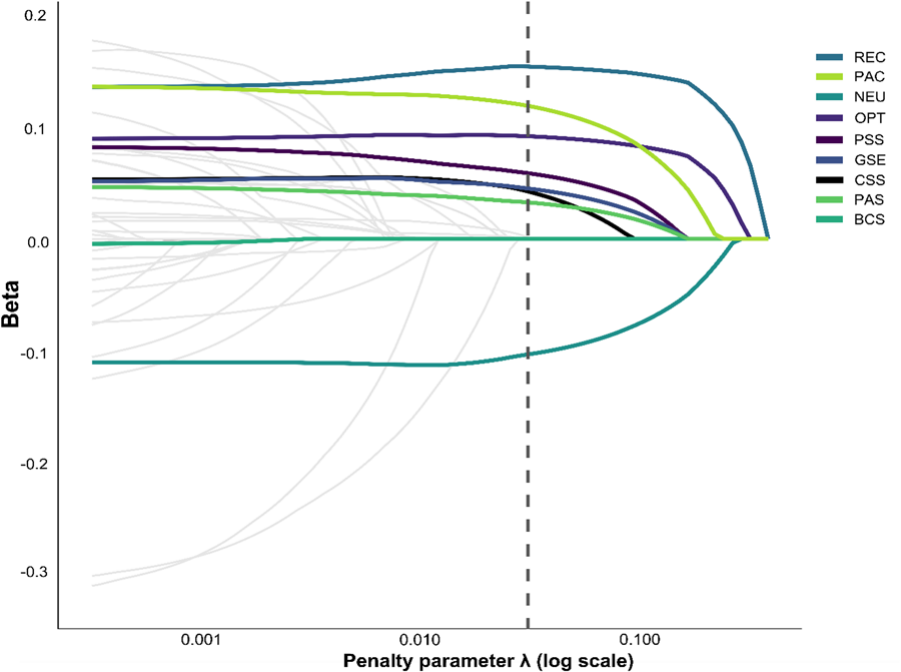
Combined multi-variable analysis (LASSO) of the relative associations of resilience factors and covariates with resilience (RES_C_). To identify the strongest of the partly correlated resilience factors, sparse regression was performed with an optimal penalty term *λ* (vertical broken line), as determined by cross-validation. Resilience factors are indicated in color, covariates in gray. The initial position of a curve on the *y*-axis signifies the association of the corresponding resilience factor or covariate with RES_C_ in the case of very low penalization. By increasing *λ* (*x*-axis), regression coefficients (β) get increasingly drawn to zero, to leave only the strongest associations. The order of resilience factors in the color legend corresponds to their determined relative strengths (absolute values) at optimal *λ* (broken line). Except for BCS (behavioral coping style), all resilience factors were selected in all 800 repeated LASSO runs, indicating strong replication stability.

Noticeable intercorrelations between resilience factors were observed for the theoretically related constructs PAS, optimism, general self-efficacy, REC, and (negatively) neuroticism (Supplementary Table S7 and Supplement 2.2.2). PAS further showed a positive relationship with positive appraisal specifically of the Corona crisis (PAC). In a context of several interrelated resilience factors, the above separate multiple regression analyses are not informative about the relative strengths of these factors in explaining RES. To determine the statistically most influential factors, we combined all variables and covariates in a regularized regression analysis (LASSO^38^) on RES_C_. This highlighted the role of REC, followed by PAC, and (negatively) neuroticism (Fig. 2). The total variance explained by all factors can be expressed in LASSO using the deviance ratio (proportion of deviance explained by regression coefficients at optimal *λ*, relative to a saturated model), which amounted to 0.23.

### Subgroup analyses

Exploratory subgroup analyses revealed significant interactions of varying subsets of the tested socio-demographic covariates with the resilience factors (bold on left side of Table 1). However, comparison of the effect estimates between levels of a covariate in Table 1 shows that, in no case, did any regression coefficient for any resilience factor change sign between covariate levels. That is, all resilience factors with a positive association with RES_C_ in the above main analysis also had positive associations at all levels of all covariates, while neuroticism always had a negative association. Their 99% confidence intervals (not shown) only crossed the zero line in 6 out of 261 cases (29 covariate levels × 9 resilience factors). Descriptively, effect sizes globally decreased with increasing household income and years of education, and were generally stronger in participants with a past or present mental health diagnosis (compare also deviance ratios on right side of the table). Further underlining the global generalizability of the effects, the LASSO analyses comparing the strengths of resilience factors within each level of each covariate showed that the relative ranks of the resilience factors did not markedly differ between covariate levels (right side of Table 1). Notable exceptions that may inform targeted interventions include a relatively reduced positive association with RES_C_ of PAC in participants above 61 years (where PAC occupied rank 6, compared to rank 2 in all other age groups) and a heterogeneous pattern of ranks between tested countries of residence. However, as our sample is not representative, as the use of questionnaires may vary between countries for linguistic or cultural reasons, and as the public health, societal, and political impacts of the pandemic may also have differed substantially between countries, we consider the country-specific result of hypothesis-generating value only and refrain from further discussion.

**Table 1:** Subgroup analyses (socio-demographic covariates).

Covariate subgroup	PAS	PSS	CSS	OPT	GSE	REC	NEU	BCS	PAC	PAS rank	PSS rank	CSS rank	OPT rank	GSE rank	REC rank	NEU rank	BCS rank	PAC rank	Deviance ratio
Gender: male	0.268	0.220	0.143	0.311	0.255	0.346	−0.319	0.081	0.223	5	6	7	2	8	1	4	9	3	0.240
Gender: female	0.253	0.241	0.136	0.300	0.263	0.333	−0.302	0.118	0.254	8	5	7	4	6	1	3	9	2	0.225
Age (years): 18–30	0.252	**0.237**	**0.099**	0.307	0.252	0.336	−0.296	0.115	**0.257**	7	**5**	**8**	3	6	1	4	9	**2**	0.205
Age (years): 31–45	0.264	**0.223**	**0.135**	0.290	0.265	0.325	−0.307	0.084	**0.239**	7	**5**	**8**	4	6	1	3	9	**2**	0.209
Age (years): 46–60	0.263	**0.267**	**0.164**	0.311	0.277	0.353	−0.325	0.141	**0.257**	7	**3**	**6**	5	8	1	4	9	**2**	0.239
Age (years): 61 +	0.230	**0.178**	**0.161**	0.301	0.218	0.351	−0.310	0.090	**0.169**	5	**7**	**4**	2	8	1	3	9	**6**	0.226
Country of residence: Belgium	**0.257**	**0.292**	**0.163**	**0.311**	**0.272**	**0.367**	**−0.302**	**0.090**	**0.215**	**3**	**2**	**6**	**5**	**7**	**1**	**4**	**8**	**9**	0.170
Country of residence: Germany	**0.206**	**0.229**	**0.106**	**0.257**	**0.255**	**0.268**	**−0.235**	**0.099**	**0.246**	**9**	**6**	**7**	**5**	**4**	**2**	**3**	**8**	**1**	0.177
Country of residence: Hong Kong	**0.228**	**0.198**	**0.158**	**0.281**	**0.183**	**0.315**	**−0.353**	**0.117**	**0.157**	**5**	**6**	**4**	**1**	**7**	**3**	**2**	**8**	**9**	0.155
Country of residence: Hungary	**0.338**	**0.313**	**0.132**	**0.291**	**0.248**	**0.360**	**−0.396**	**0.207**	**0.306**	**5**	**3**	**6**	**7**	**8**	**2**	**1**	**9**	**4**	0.211
Country of residence: Italy	**0.275**	**0.239**	**0.089**	**0.318**	**0.198**	**0.390**	**−0.314**	**0.105**	**0.261**	**5**	**6**	**7**	**2**	**8**	**1**	**4**	**9**	**3**	0.167
Country of residence: The Netherlands	**0.334**	**0.357**	**0.249**	**0.393**	**0.373**	**0.439**	**−0.351**	**0.157**	**0.274**	**7**	**2**	**3**	**5**	**6**	**1**	**8**	**9**	**4**	0.325
Country of residence: Poland	**0.378**	**0.171**	**0.157**	**0.344**	**0.276**	**0.437**	**−0.456**	**0.128**	**0.292**	**4**	**7**	**6**	**3**	**8**	**1**	**2**	**9**	**5**	0.278
Country of residence: Serbia	**0.251**	**0.133**	**0.100**	**0.215**	**0.261**	**0.248**	**−0.296**	**0.131**	**0.225**	**3**	**7**	**6**	**5**	**2**	**8**	**1**	**9**	**4**	0.145
Country of residence: Other	**0.201**	**0.198**	**0.079**	**0.303**	**0.222**	**0.308**	**−0.302**	**0.071**	**0.202**	**9**	**6**	**7**	**2**	**5**	**3**	**1**	**8**	**4**	0.169
H.h. income: 0–4999€	**0.302**	0.244	0.144	0.330	0.250	**0.386**	**−0.358**	**0.133**	0.249	**5**	6	7	2	8	**1**	**3**	**9**	4	0.194
H.h. income: 5000–9999€	**0.265**	0.254	0.128	0.279	0.287	**0.391**	**−0.391**	**0.192**	0.270	**7**	4	8	6	5	**1**	**2**	**9**	3	0.250
H.h. income: 10,000–14,999€	**0.301**	0.216	0.157	0.335	0.244	**0.349**	**−0.337**	**0.124**	0.303	**5**	7	6	2	8	**3**	**4**	**9**	1	0.227
H.h. income: 15,000–24,999€	**0.290**	0.229	0.119	0.335	0.249	**0.365**	**−0.332**	**0.095**	0.254	**5**	6	7	2	8	**1**	**4**	**9**	3	0.225
H.h. income: 25,000–49,999€	**0.244**	0.234	0.146	0.289	0.281	**0.333**	**−0.294**	**0.100**	0.237	**8**	7	6	5	4	**1**	**3**	**9**	2	0.223
H.h. income: 50,000–74,999€	**0.210**	0.234	0.119	0.277	0.258	**0.298**	**−0.256**	**0.116**	0.209	**9**	4	7	3	5	**1**	**6**	**8**	2	0.178
H.h. income: 75,000–99,999€	**0.212**	0.219	0.108	0.291	0.236	**0.300**	**−0.270**	**0.082**	0.208	**9**	5	7	3	6	**1**	**4**	**8**	2	0.187
H.h. income: 100,000+ €	**0.239**	0.236	0.128	0.270	0.229	**0.286**	**−0.273**	**0.052**	0.216	**7**	5	8	2	6	**1**	**3**	**9**	4	0.166
Education (years): 0–12	**0.248**	0.242	0.158	**0.319**	0.267	**0.371**	**−0.356**	0.113	0.232	**9**	5	7	**3**	6	**1**	**2**	8	4	0.224
Education (years): 13–16	**0.296**	0.244	0.128	**0.337**	0.279	**0.368**	**−0.346**	0.122	0.248	**5**	6	8	**2**	7	**1**	**3**	9	4	0.257
Education (years): 17–20	**0.241**	0.234	0.143	**0.284**	0.248	**0.322**	**−0.294**	0.109	0.244	**8**	5	6	**4**	7	**1**	**3**	9	2	0.209
Education (years): 21+	**0.219**	0.201	0.101	**0.263**	0.229	**0.303**	**−0.243**	0.080	0.236	**9**	5	7	**3**	4	**1**	**6**	8	2	0.167
Mental health diagnosis: yes	**0.316**	**0.277**	0.148	**0.346**	**0.315**	**0.402**	**−0.351**	**0.184**	**0.311**	**7**	**5**	8	**3**	**4**	**1**	**6**	**9**	**2**	0.243
Mental health diagnosis: no	**0.227**	**0.203**	0.126	**0.272**	**0.219**	**0.309**	**−0.283**	**0.082**	**0.220**	**7**	**6**	5	**4**	**8**	**1**	**3**	**9**	**2**	0.199

*H.h.* household.

Left side: regression coefficients for each subgroup level from linear regression interaction models calculated separately for each resilience factor (column). Statistics associated with subgroup levels of a covariate that significantly interacted with the respective resilience factor are highlighted in bold. Right side: ranks indicating the relative strengths of resilience factors at the respective subgroup level, derived from their order of magnitude at optimal λ in LASSO analyses.

## Discussion

We identify a positive association between resilience—defined as the outcome of maintained good mental health during a 2 weeks’ period of stressor exposure that fell into the most intense phase of the Corona lockdown in Europe—and PAS. This finding confirms and extends yet unpublished findings from our longitudinal studies that are being conducted in populations of healthy adults confronted with general stressors of everyday life, but not with situations comparable to the Corona crisis (Supplement 2.2.2). We also identify positive associations between other hypothesized resilience factors and resilience. As our hypotheses are nearly exclusively derived from analyses of populations confronted with stressors other than a pandemic, this indicates that many of the resilience factors described so far^27,29,30^ may be “global”^31^, i.e., protective in different types of adverse circumstances. This result was not expected because we had earlier theorized that different circumstances and stressors likely require different adaptive psychological and behavioral responses in order to not damage mental health^31^. However, the generalizability of resilience factors is also supported by our exploratory subgroup analyses, which show globally consistent effects across major socio-demographic categories, including individuals with a past or present mental health diagnosis. These findings raise hopes that existing techniques for enhancing known resilience factors (e.g., see refs. ^44,45^), may also be effective in pandemics and, more generally, may be of use in any or most types of adversity and populations. This global statement must be moderated by the observation of apparently reduced effect sizes for the tested psycho-social resilience factors (but also of the vulnerability factor neuroticism) in wealthier and more educated participants and the weaker role for positive appraisal in the oldest participants. These groups may partly rely on other sources for coping that we could not identify in this study. Conversely, individuals with past or present diagnosed mental health problems may be more reliant on psycho-social factors.

In a comparative analysis of resilience factors (LASSO; Fig. 2), we identify REC and PAC as the two most important factors. Interestingly, PAC outperformed PAS as well as the related constructs optimism and general self-efficacy (Supplement 2.2.2 and ref. ^31^). Our PAC instrument asks participants about their current estimates of the consequences of the crisis for themselves and for society. This suggests that measuring the appraisal of the dominant stressors in a given situation has even better potential to explain their RES than measuring a general appraisal style or tendency (PAS) or also tendencies in the appraisal of specific threat dimensions, such as threat probability (generally appraised as low in individuals with high optimism) or coping potential (generally appraised as high in individuals with high self-efficacy).

The REC instrument used in our survey questionnaire^46^ asks questions about typical, trait-like stress reactions. Thereby, it is semantically close to our mental health instrument, used to calculate RES, which asks questions about current, symptom-like stress reactions^33^. The semantic closeness of the instruments may explain the strong statistical relationship between REC and RES_C_, and places the REC construct somewhere between predictor and outcome variable.

This is interesting in the context of PASTOR^31^. PASTOR claims that the common final pathway to maintain mental health in the face of adversity lies in the tendency to appraise potential stressors with a.o., an optimistic perspective on the probability of bad outcomes of the threatening situation and under the assumption of a high coping potential in case of a bad outcome (including high self-efficacy expectations; hence, the observed relationships with optimism and self-efficacy in Supplementary Table S7). At the same time, positive appraisal in the sense of PASTOR avoids extremely unrealistically positive (delusional) appraisal tendencies that might give rise to trivialization or blind optimism. Positive appraisal effectively fine-tunes stress responses to optimal levels, that is, it produces stress reactions when necessary but also avoids unnecessarily strong, prolonged, or repeated stress reactions. This prevents inefficient deployment of resources and concomitant deleterious allostatic load effects and reduces the likelihood of developing stress-related mental problems^31^.

The notion that positive appraisal permits optimal stress responding leads to the hypothesis that individuals showing high PAS scores have stress responses that are not higher and especially not longer than necessary (i.e., good stress response recovery). It is through this pathway that positive appraisal eventually results in maintained mental health despite stressor exposure (i.e., resilience)^31^. Our finding that REC statistically partly mediates the relationship of PAS with RES is in agreement with this hypothesis and may be another explanation for the close statistical relationship between REC and RES.

A further aspect of PASTOR is worth noting. By positing that positive appraisal is the common final pathway to mental health (mediated by optimal stress responding), PASTOR also posits that the effects of other, especially non-cognitive resilience factors, are mediated by their way they shape PAS. That is, other resilience factors are more distal to the outcome of resilience relative to the proximal factor PAS. An explicit example given in 31 is the expected mediation of the effect of perceived social support on RES by PAS, based on the assumption that believing that one can rely on others will make potential stressors be perceived as generally less threatening. Our results also agree with this second mediation hypothesis and therefore yield initial support for the theory.

Our findings identify psychological constructs that are promising targets of measures to protect mental health during pandemics. PAS/PAC may be of specific interest both because of their proximal position relative to the resilient outcome and because positive appraisal tendency is specifically conceived as a malleable individual property that has some stability (hence, “style”) but can also be changed by experience and training^31^. Further, a key element in cognitive behavioral therapy and related evidence-based psychotherapy techniques is to change maladaptive threat appraisals^47^. This suggests an effective approach in pandemics or other crises may be to change potentially unhelpful appraisal patterns towards a more productive attitude. This can be achieved through individual remote counseling or therapy, including via hotlines, (internet-based) provision of self-help materials and courses, suitable computer or smartphone apps, care in individual and public communication, and the generation of appropriate media content^48–52^. (See also adaa.org/finding-help/coronavirus-anxiety-helpful-resources as an example.) An appraisal-focused approach does not preclude approaches targeting other resilience factors, such as social support^44,51^.

A limitation of our study is that we are unable here to provide longitudinal data, which are considered the gold standard in resilience research^27^. By contrast, in our study, changes in mental health over the past 2 weeks are assessed retrospectively and therefore potentially affected by memory biases. Further, the associations we report are based on assessments conducted at the same time point, rather than being longitudinal, which may lead to overestimation of effects^53^. Therefore, our results will have to be confirmed by an ongoing longitudinal study with the same questionnaire (www.dynacore.info), which will, however, only yield final results in several months. Another limitation is that our sample is self-selected and may thus not be representative (see also Supplementary Table S1). Our conclusions are therefore of mechanistic nature—we identify effective resilience factors, but we cannot claim that they are effective in the general population or in specific subgroups of the population. Finally, we have emphasized earlier that an approach to measure positive appraisal style with self-report instruments has the disadvantage that self-report cannot inform about appraisal contents or processes that are not accessible to consciousness or not verbalizable, and that self-report has principle problems related to the quantification of introspective qualia, semantic ambiguity, and socially desirable reporting^31^. Efforts to supplement or replace our self-report method with more objective instruments are ongoing.

To conclude, a resilience-focused approach to the psychological consequences of the Corona pandemic identifies protective factors that can be leveraged in efforts to prevent likely negative mental health consequences of the current crisis.

## Supplementary information

Supplement

## Data Availability

The anonymous data (cleaned complete and incomplete data sets) are available at osf.io/5xq9p/.

## Code availability

The scripts for data cleaning and analysis are available at osf.io/5xq9p/.
