# Supplementary material for: Psycho-social factors associated with mental resilience in the Corona lockdown
Source: Transl Psychiatry. 2021 Jan 21;11:67. doi: 10.1038/s41398-020-01150-4 (PMC7817958; doi:10.1038/s41398-020-01150-4)
Supplement: Supplementary file 1 — Supplement [file 41398_2020_1150_MOESM1_ESM.pdf]

Supplement to

Psycho-social factors associated with mental resilience in the Corona lockdown

by Veer et al.

A description of the methods and the hypotheses is also available in the preregistration of the first interim analysis (osf.io/r6btn) and in the amendment to that preregistration, describing the second interim analysis (osf.io/thka9).

Table of contents

1. Survey questionnaire
2. Derivation of hypotheses and outcome measures
3. Supplementary Tables
   - Table S1: Sample characteristics (N=15,790 complete valid data sets).
   - Table S2: Questionnaire elements: psychological constructs and used instruments.
   - Table S3: Variables, indices and covariates.
   - Table S4: Past or present diagnosed mental health conditions.
   - Table S5: A priori hypotheses.
   - Table S6: Assessed stressors, stressor counts and average severity ratings.
   - Table S7: Correlations of independent variables (hypothesized resilience factors) and outcome-based resilience (RES)
   - Table S8: Tests of directed hypotheses H1-H9 (outcome: resilience to all stressors combined, RES_C)
   - Table S9: Tests of directed hypotheses H1-H9 (outcome: resilience to general stressors, RES_G)
   - Table S10: Tests of directed hypotheses H1-H9 (outcome: resilience to Corona crisis-specific stressors, RES_S)
4. Incomplete data
5. References

1 Survey questionnaire

"DynaCORE-C - the DynaMORE cross-sectional survey study on psychological resilience to the mental health consequences of the Corona crisis" was conducted by the EU project DynaMORE (Dynamic MOdelling of REsilience), see www.dynamore-project.eu. Study participation was possible at bit.ly/DynaCORE-C or www.dynacore.info. At the time when data were pulled for the second interim analysis reported in this paper (April 20th 2020), available languages were Arabic, Chinese, Croatian, Czech, Danish, Dutch, English, Estonian, Finnish, French, German, Greek, Hebrew, Hungarian, Italian, Lithuanian, Norwegian, Polish, Portuguese, Serbian, Slovak, Spanish, Swedish, and Ukrainian. The 127-item survey questionnaire contains a combination of self-generated questions and questions from existing instruments (see Table S2). In some instances, instruments were shortened or instructions or questions were adapted to better fit the study context. The quality of translations from the original English version into other languages was assured by using back-translation by independent translators. Where available, local published validated versions of the instruments included in the questionnaire were used. The questionnaires are privately stored in osf.io/5xq9p/ and are made available upon request. Before the final form of the questionnaire was reached, near-identical prefinal versions were already employed in which answer options to the household income question were less fine-grained (the lowest range "0 to 24.999 €" was overly broad: first 355 complete valid data sets, of whom 346 from European residents) and in which previous or current psychiatric disorder was not enquired (first n=406 complete valid data sets, or n=512 complete European data sets that were partially invalid due to the prefinal income range options).

2 Derivation of hypotheses and outcome measures

2.1 General note on resilience

We here base our efforts to gain insight into protective factors in the Corona crisis on a resilience framework (Kalisch et al., 2017). The science of resilience is based on the well-documented observation that some individuals maintain mental health despite exposure to severe psychological or physical adversity (Bonanno and Mancini, 2011; Kalisch et al., 2015). Resilience research aims to understand why some people do not (or only temporarily) develop stress-related mental dysfunction, despite being subject to the same kind of challenges that cause long-term dysfunction in others. This approach is naturally linked to the question of how to prevent stress-related mental health conditions, rather than attempting to treat them at a later stage when significant individual suffering and societal and economic costs have already occurred (Sapienza and Masten, 2011). Hence, resilience research is health- and prevention-focused and, thereby, an alternative approach to classical disease-focused research in the battle against stress-related disorders (Kalisch et al., 2015). Prevention is one of the main under-researched and under-exploited strategies in current efforts to promote mental health (Jorm et al., 2017) and has been highlighted as one key area of research and public health measures in the European roadmap for mental health (http://www.roamer-mh.org/files/DocRoamer_Roadmap2015_FINALISSIMA_050615.pdf).

There is growing consensus that psychological (individual) resilience is best defined as the maintenance or quick recovery of mental health during and after times of adversity, which may consist in a potentially traumatizing event, challenging life circumstances, a critical life transition phase, or physical illness (Kalisch et al., 2017). Hence, resilience is conceptualized as an outcome (maintained mental health). Resilient outcomes are partly determined by pre-existing factors or predispositions, such as (trait-like as well as more modifiable and time-variant style-like) characteristics of the individual, individual resources, or features of the individual's environment. These "resilience factors" are incompletely understood, and it is unclear if factors that are protective against one type of adversity are also protective against other types of adversity. It is also unclear if resilience factors that are protective in one population (culture, nation, ethnicity, gender, social class, profession etc.) are also protective in other populations. Finally, different resilience factors may protect differentially against different types of mental health problems (anxiety, depression, compulsive symptoms, …) and such types of problems may occur at different rates in different populations during the Corona crisis (Kalisch et al., 2015, 2017). This makes it highly relevant to rapidly collect reliable information on effective resilience factors in a maximum possible number of subjects and populations world-wide in the current global crisis.

2.2 Hypothesized resilience factors (independent variables)

Despite uncertainties about effective resilience factors in the Corona pandemic, existing knowledge about resilience factors from a large variety of different types of individual-level and society-level adversities and crises can be used to formulate testable hypotheses and thereby reduce the search space.

2.2.1 Described resilience factors

We hypothesized that the described resilience factors perceived social support, optimism, and perceived general self-efficacy (Bonanno et al., 2015) will be positively associated with outcome-based resilience (in short, 'resilience' in the further). We also hypothesized that a perceived increase in social support during the crisis will be positively associated with resilience. We further hypothesized that perceived good stress recovery, as assessed by a questionnaire that asks subjects about how quickly and easily they recover from stress responses (Smith et al., 2008), will be positively associated with resilience. See Table S5 for summary.

2.2.2 New potential resilience factors: positive appraisal and behavioral coping

To these established resilience factors we added two new, related factors emanating from current, yet unpublished own research. Both factors are inspired by positive appraisal style theory of resilience (PASTOR; Kalisch et al., 2015) and are showing predictive power for resilience in two on-going longitudinal studies, LORA (LOngitudinal Resilience Assessment, www.lora.studie.de; conducted by the University Medical Center of Johannes Gutenberg University in Mainz, Germany, and the University Hospital of Goethe University in Frankfurt,

Germany; funded by Deutsche Forschungsgemeinschaft (DFG) Collaborative Research Center CRC1193 “Neurobiology of Resilience”; PIs: K. Lieb, Mainz, and A. Reif, Frankfurt; co-PIs: R. Kalisch, O. Tüscher, M. Wessa, Mainz, and U. Basten, C. J. Fiebach, M. Plichta, Frankfurt; N at inclusion: 1200; age at inclusion: 18-50 years) and MARP (MAinz Resilience Project, www.marp-studie.de; by the University Medical Center Mainz and the Leibniz Institute for Resilience Research in Mainz; funded by the State of Rhineland-Palatinate, Germany; PI: R. Kalisch; co-PIs: K. Lieb, O. Tüscher, M. Wessa; N at inclusion: 200; age at inclusion: 18-21 years). For overview and first methodological publications, see Kampa et al. (2018, 2020) and Kalisch et al. (2020).

As briefly outlined in the main text, PASTOR claims that the common final pathway to maintained mental health in the face of adversity (i.e., resilience) lies in the positive appraisal of potential stressors (threats to one’s goals and needs). ‘Positive appraisal’ sets the values that an individual attributes to a stressor on the key threat appraisal dimensions threat magnitude or cost, threat probability, and coping potential to levels that realistically reflect the threat or even slightly underestimate it. That is, positive appraisal avoids catastrophizing (magnitude/cost dimension), pessimism (probability dimension) and unnecessarily low self-efficacy or control perceptions (coping dimension). At the same time, positive appraisal avoids unrealistically positive (delusional) threat perceptions that might lead to trivialization or blind optimism. Such mildly positive appraisal permits the organism to fine-tune stress responses to optimal levels, producing stress reactions when necessary but avoiding unnecessary stress, inefficient deployment of resources and concomitant deleterious allostatic load effects. PASTOR assumes that an individual usually appraises similar situations in a similar fashion, and therefore can be characterized by his or her typical appraisal tendencies (‘appraisal style’). A negative appraisal style (NAS) consists in a propensity to overestimate the aversive consequences and the probability of challenging situations and to underestimate one’s coping potential. An NAS results in consistent over-reactions to threats and increases the likelihood of an individual developing stress-related mental problems during and after adversity. A positive appraisal style (PAS), by contrast, is defined as the absence of such negative biases, but also by the absence of delusional positive appraisal tendencies. A PAS typically results in realistic threat estimations or mild underestimations of threat and is claimed to be protective for mental health. Hence, PAS includes the constructs of optimism and general self-efficacy, but is broader (Kalisch et al., 2015).

Importantly, a PAS is believed to be determined by the efficacy and efficiency of the neural and cognitive processes that underlie positive stressor appraisal (that produce appraisals when subjects are challenged). This opens two different roads towards assessing appraisal style with self-report instruments: one can assess the typical appraisal contents that an individual produces in response to stressors, and one can assess the typical thinking processes, or mental operations, that individuals perform when they are challenged. An example for the former would be that a subject reports to see a positive aspect in a threatening situation or to find that there are worse things in life. An example of the latter would be that a subject reports to usually try to think of positive aspects of a situation or to try to tell oneself that there are worse things in life. Everyday language often does not clearly differentiate between appraisal contents and processes, and also pre-formulated questions in existing questionnaire instruments are often a mix of more content- and more process-focused propositions.

In MARP and LORA, we employ two established instruments developed to capture various behavioral and cognitive coping and emotion regulation strategies and that include, among others, questions assessing various appraisal contents and processes, often in a mixed fashion: the brief COPE (Carver, 1997) and the CERQ-short (Garnefski and Kraaij, 2006). The brief COPE has 14, the CERQ-short has nine two-item subscales. In addition, we amended the CERQ-short with two own-formulated questions on a distanced (detached) stressor appraisal. In the baseline (T0) data from both samples, we conducted a factor analysis on the subscale-level including both questionnaires and (in MARP) the additional distancing subscale. This reliably identified only three factors spanning the questionnaires. Several subscales loaded predominantly on one of the three factors, such that they could be qualified as factor-characteristic subscales. Of these, the subscales distancing (dis; own-formulated), positive reappraisal (pra; CERQ), acceptance (acc; CERQ, less COPE), putting into perspective (per; CERQ), positive reframing (posref; COPE), and – to a lesser extent - refocus on planning (rfp; CERQ), positive refocusing (prf; CERQ) and humor (hum; COPE) strongly positively loaded on the first factor. Another factor was dominated by less cognitive, more behavioral coping

subscales, namely instrumental support seeking (iss), emotional support seeking (ess), venting of emotions (vent), and acting out (act), all from the COPE. Both factors were positively predictive of resilience (outcome-based, for details see below), though the first more so. Detailed results from this factor analysis and the MARP and LORA studies will be published elsewhere.

We interpret the first, cognitive factor as reflecting positive appraisal contents and processes and therefore consider it an appropriate self-report measure of PAS. For the current project, we dropped the posref and acc subscales from the COPE, given that they conceptually overlap highly with pra and acc from the CERQ but loaded less on the first factor. This left the seven subscales dis, pra, acc, per, rfp, prf, and hum, which we henceforth refer to as PASS, for ‘positive appraisal style scale’. We confirmed that a sum score from these subscales also positively predict resilience in MARP and LORA. We hypothesize that PASS will be positively associated with resilient outcome in the current project.

Because of a (lesser) positive association of the behavioral coping factor with resilience in MARP and LORA, we also include the eight questions for iss, ess, vent and act, which together we here term BCS, for ‘behavioral coping scale’. Behavioral coping may also be helpful in the Corona crisis, and we therefore also hypothesize that BCS will be positively associated with resilience.

Another, yet unpublished result from MARP is that personality instruments including the PANAS (Watson et al., 1988) and the BFI-10 (Rammstedt and John, 2007) could be summarized factor-analytically in one common factor that reflects a positive vs. a negative trait affect (Jeronimus et al., 2016; Schenk et al., 2018). The factor was positively loaded by positive affect items from the PANAS and highly negatively by the neuroticism subscale of the BFI-10 and also predicted resilience. For brevity, we here selected two neuroticism items and hypothesize that their sum score will be negatively associated with resilience. Because in MARP and LORA, PASS and neuroticism are negatively correlated, they may reflect overlapping constructs that could be summarized as a general positive emotional style.

Finally, we hypothesize that the positive appraisal specifically of the current pandemic will be positively associated with resilience. To test this, two custom-made questions were generated. (‘I expect that I will learn something positive from of the Corona pandemic for my own life.’ and ‘In the long run, I think that society will change for the better because of the Corona pandemic.’)

The survey questionnaire also contained room for open answers, for hypothesis generation about resilience factors. Results from this analysis will be reported elsewhere.

2.2.3 Mediation effects

Based on Kalisch et al. (2015) (PASTOR), we hypothesize that the expected positive effect of social support on resilience is positively mediated by its effect on PAS. PASTOR further claims that positive appraisal permits the organism to fine-tune stress responses to optimal levels, thus avoiding unnecessary stress. This includes having stress responses that are not higher in magnitude and especially not longer in duration than necessary. It is through this pathway that positive appraisal eventually results in maintained mental health despite stressor exposure (i.e., resilience). This response pattern of usually only having quickly recovering stress responses is assessed by the Brief Resilience Scale (BRS; Smith et al., 2008). It can therefore be hypothesized that the effect of PAS on resilience is positively mediated by its effect on self-perceived good stress recovery.

The preregistered hypotheses (osf.io/r6btn) are summarized in Table S5.

2.3 Resilience outcome measures (dependent variables)

Our outcome-based definition of resilience as maintenance or quick recovery of mental health during and after adversity (Kalisch et al., 2017; see 2.1) implies that adversity is necessarily part of the equation. Only registering mental health outcomes without taking into account the adversity a subject was or is exposed to may be informative about mental health, but is not informative about resilience, which in its essence is mental health despite adversity (Mancini and Bonanno, 2009; Kalisch et al., 2017). Ideally, this is achieved by measuring mental health problems (P) before (T0) and after (T1) some time of stressor exposure (E) and normalizing changes in mental health between T0 and T1 by the stressor exposure that has occurred in between (Kalisch et al., 2015, 2017). In such a scenario, of two individuals having experienced

comparable stressor exposure E, the individual with fewer increases in mental health problems P would be the more resilient individual. Another scenario would be two individuals showing similar increase in P with different E. Here, the individual with higher E would be the more resilient one. A detailed description of how we measure mental health and stressors in the longitudinal studies MARP and LORA and use those data to calculate resilience outcomes is available in Kalisch et al. (2020).

2.3.1 Mental health measure

For practical reasons, the current study uses a cross-sectional design. We can thus not perform repeated mental health assessments. We therefore make use of a specific feature of our mental health instrument, the short 12-item version of the General Health Questionnaire (GHQ-12; Goldberg et al., 1997), which is designed to ask subjects about how they felt in the past two weeks relative to how they normally feel. Thus, the GHQ assesses a perceived change in mental health, which we will use as an approximation of a measured difference in mental health between two time points. See Table S3 for scoring of the GHQ, to derive the mental health problem score P.

2.3.2 Stressor exposure measures

For stressor exposure, we employ two instruments as part of the survey questionnaire. The first instrument is a list of eleven categories of daily hassles (DHs) and life events (LEs), which we condensed specifically for this study from the much more extensive DH (58 items) and LE (27 items) lists employed in MARP and LORA (general stressor exposure instrument, for calculation of stressor exposure score E_G). We were mainly guided by the frequency of DHs reported at study inclusion by the LORA participants, which is the larger and more age-representative sample than MARP (N=1200 at inclusion, age range at inclusion 18-50 years). The most frequently reported DHs in LORA were household chores, commuting, bad weather, interruption in activities, performance pressure, negative events in the media, time pressure, and waiting time or delay. The second instrument employed in the current study to assess stressors is a list of 29 stressors that we collated to reflect situations of adversity related to the Corona pandemic (Corona pandemic-specific stressor exposure, for calculation of stressor exposure score E_S). In both instruments, subjects are requested to indicate whether they experience a given stressor currently or have experienced it during the last two weeks. If they report to have experienced the stressor, they are further asked to report how burdensome it was to them (severity rating). (In addition, for both E_G and E_S, respondents have the possibility to add and rate one stressor that was not mentioned in our predefined lists.) In this way, we obtain an overview of recent stressor exposure E roughly in the time period across which subjects also report changes in P, using the GHQ. We can then meaningfully relate both variables to capture stressor-related changes in mental health.

To derive E from participants' responses, we here use two alternative scoring approaches. In the first approach, we simply count the reported listed stressors (stressor count method); in the second approach, we weigh each reported stressor (including 'other') by its severity rating and form a weighted sum (stressor severity method). The advantage of the stressor count method is that it restricts E to reflect the mere occurrence of stressors, not being confounded by individual differences in the way stressors are perceived (appraised). We use this scoring method in MARP and LORA, where we have long stressor lists (85 items) and can thus assume that the mere frequency of stressors is a good reflection of objective stressor exposure E. Factual differences in the severity of a given stressor can be ignored, given the sheer number of reportable stressors (Chmitorz et al., 2020). This assumption may hold only partly for the current study, which uses a shorter list of only 40 stressors. The advantage of the stressor severity method is that factually (objectively) strong individual stressors are appropriately taken into account. For instance, own serious health problems due to a COVID-19 infection or a job loss that threatens material survival may be objectively highly burdensome and will be taken into account as such with the severity method. The downside is that subjective over-reactions (i.e., overly negative appraisal) may inflate the E measure. This is a relevant potential confound, because relating a change in P to an inflated E measure may lead to an overestimation of a subject's resilience.

Another question regarding E scoring is whether general and Corona pandemic-specific stressors should be combined, to derive a combined stressor exposure score E_C and, consequentially, to determine subjects' resilience to all stressors; or whether E_G and E_S should

be treated separately. In our preregistered analysis plan, we leave this question as well as the question of the optimal scoring method open and plan to answer them based on data, using below decision rule (see 2.3.3).

To summarize, our data allow us to calculate six different stressor exposure scores:

- a general stressor exposure score based on the stressor count method ($E_{G,SCM}$),
- a general stressor exposure score based on the stressor severity method ($E_{G,SSM}$),
- a Corona pandemic-specific stressor exposure score based on the stressor count method ($E_{S,SCM}$),
- a Corona pandemic-specific stressor exposure score based on the stressor severity method ($E_{S,SSM}$),
- a combined stressor exposure score based on the stressor count method ($E_{C,SCM}$),
- a combined stressor exposure score based on the stressor severity method ($E_{C,SSM}$).

2.3.3 Relationships between mental health and stressor exposure measures

In MARP and LORA, we observe predominantly monotone E-P relationships in the range of R=0.3 to 0.4. This applies to both scoring methods and to combined as well as DH- and LE-specific E scores. The strongest E-P relationship is consistently observed in both samples for combined stressor exposure E_C, calculated based on the theoretically preferred stressor count method (see 2.3.2), which is why we use this score as a basis for calculating resilience outcomes in MARP and LORA (Kalisch et al., 2020). We expected similar E-P relationships in the current study. As in MARP and LORA, we therefore explored which E score explains most variance in P and based our main resilience measure on this score. This applied to $E_{C,SSM}$ ($E_{C,SSM}$: R^2=0.21; $E_{G,SSM}$: R^2=0.17; $E_{S,SSM}$: R^2=0.18; comp. $E_{C,SCM}$: R^2=0.07; $E_{G,SCM}$: R^2=0.08; $E_{S,SCM}$: R^2=0.04). We had defined in the preregistered analysis plan (osf.io/r6btn): "If the chosen E score happens to combine general and Corona pandemic-specific stressors, we will subsequently compute separate analyses for both stressor categories, to also learn about potential differential effectiveness of resilience factors for each category. We will use the same scoring method (either count or severity) for these secondary scores as for the chosen primary score." This strategy is realized in the main text ($E_{C,SSM}$, followed by $E_{G,SSM}$ and $E_{S,SSM}$). We further defined: "If the E-P relationship turns out not to be predominantly linear, the decision will be made based on the predominant form of relationship (e.g., quadratic)." We observed that a model with linear and quadratic effects had a much better model fit (RES_C: F=556.68; RES_G: F=637.82; RES_S: 353.78; all p<0.001) than a model with a linear effect only (comp. van Harmelen et al., 2017) and therefore used the former to calculate individual RES scores.

2.3.4 Calculation of resilience outcome measures based on mental health and stressor exposure measures

Based on the expected observation of a robust linear E-P relationship and on above decision on the chosen E score ($E_{C,SSM}$), the distance of an individual's P score to the E-P regression line can be considered informative about the reactivity of his/her mental health to his/her stressor exposure. The regression line is viewed as the normative reactivity of mental health to stressor exposure (in short: 'stressor reactivity', SR) in the whole sample. A subject's residual onto the regression line expresses to what extent the subject deviates from that normal E-P relationship. Individuals with positive residual values show "too many" mental health problems, given their level of stressor exposure; individuals with negative values show "too few" mental health problems, given their stressor exposure (ignoring random variability for the moment). In other words: a positive residual reflects over-reactivity of mental health to stressor exposure (high stressor reactivity, high SR); a negative residual reflects under-reactivity (low SR) (Kalisch et al., 2020).

The advantage of using residualization is that the method inherently corrects for individual differences in the level of stressor exposure. Two subjects 1 and 2 that have developed comparable mental health problems P over the last two weeks may still diverge in their SR score, for instance, if subject 1 had higher exposure E than subject 2. Subject 1 would then obtain a lower SR score and could be classified as more resilient than subject 2, in accordance with our definition of resilience as an outcome (Kalisch et al., 2015, 2017), that is, as mental health maintenance despite stressor exposure. Two subjects 3 and 4 with comparable stressor exposure E would also obtain different SR scores, for instance, when 3 has developed fewer

problems P than subject 4. Subject 3 would then obtain a lower SR score and could be regarded more resilient. On this basis, we defined the inverse of SR as our outcome-based resilience measure RES (Kalisch et al., 2020).

Residualization approaches have been introduced into the resilience literature by Amstadter et al. (2014) and van Harmelen et al. (2017).

A potential criticism of the SR score might be that they are also calculated for individuals with very low stressor exposure E, and that resilience in the absence of significant adversity is not a meaningful concept (Mancini and Bonanno, 2009). We therefore adopted as a general rule for the analyses of SR data that a) primary analyses based on the entire sample be complemented by secondary analyses of those two thirds of the subjects with the highest overall stressor exposure E, and that b) the results of those secondary analyses should go in the same direction as those of the primary analyses, in order for the primary analysis results to be considered valid (Kalisch et al., 2020). All results of these secondary analyses went into the same direction as those of the primary analyses (not shown).

3 Supplementary Tables

	Overall	Male	Female	Diverse
	(N=15790)	(N=4209, 26.7%)	(N=11439, 72.4%)	(N=142, 0.9%)
**Age (years)**				
Mean (SD)	40.0 (14.4)	41.2 (15.3)	39.7 (14.0)	31.8 (12.4)
Median [Min, Max]	38.0 [18.0, 91.0]	39.0 [18.0, 91.0]	38.0 [18.0, 84.0]	27.0 [18.0, 74.0]
18-30	5166 (32.7%)	1315 (31.2%)	3764 (32.9%)	87 (61.3%)
31-45	5186 (32.8%)	1334 (31.7%)	3820 (33.4%)	32 (22.5%)
46-60	3846 (24.4%)	997 (23.7%)	2831 (24.7%)	18 (12.7%)
61 +	1592 (10.1%)	563 (13.4%)	1024 (9.0%)	5 (3.5%)
**Belonging to a risk group for severe or life-threatening COVID-19 symptoms (N)**				
yes	2056 (13.0%)	613 (14.6%)	1429 (12.5%)	14 (9.9%)
no	10908 (69.1%)	2781 (66.1%)	8031 (70.2%)	96 (67.6%)
unsure	2826 (17.9%)	815 (19.4%)	1979 (17.3%)	32 (22.5%)
**Tested positive for COVID-19 (N)**				
yes	35 (0.2%)	12 (0.3%)	23 (0.2%)	0 (0%)
no	15755 (99.8%)	4197 (99.7%)	11416 (99.8%)	142 (100%)
**Quarantine (N)**				
none	14199 (89.9%)	3799 (90.3%)	10280 (89.9%)	120 (84.5%)
at home	1423 (9.0%)	371 (8.8%)	1034 (9.0%)	18 (12.7%)
in the hospital	5 (0.0%)	2 (0.0%)	2 (0.0%)	1 (0.7%)
abroad	26 (0.2%)	7 (0.2%)	18 (0.2%)	1 (0.7%)
other	137 (0.9%)	30 (0.7%)	105 (0.9%)	2 (1.4%)
**Agreement with authorities' measures to curtail the spread of the Corona virus**				
Mean (SD)	3.85 (1.18)	3.72 (1.26)	3.89 (1.15)	3.63 (1.21)
Median [Min, Max]	4.00 [1.00, 5.00]	4.00 [1.00, 5.00]	4.00 [1.00, 5.00]	4.00 [1.00, 5.00]
**Following recommended procedures to limit the spread of the Corona virus**				
Mean (SD)	4.54 (0.716)	4.44 (0.794)	4.57 (0.677)	4.39 (0.995)
Median [Min, Max]	5.00 [1.00, 5.00]	5.00 [1.00, 5.00]	5.00 [1.00, 5.00]	5.00 [1.00, 5.00]
**Past or present diagnosed mental health condition**				
yes	3607 (22.8%)	711 (16.9%)	2820 (24.7%)	76 (53.5%)
no	11777 (74.6%)	3376 (80.2%)	8337 (72.9%)	64 (45.1%)
not asked	406 (2.6%)	122 (2.9%)	282 (2.5%)	2 (1.4%)
**Good general health (self-report, 1-5)**				
Mean (SD)	2.55 (1.01)	2.68 (1.03)	2.51 (1.00)	2.51 (1.14)
Median [Min, Max]	2.00 [1.00, 5.00]	3.00 [1.00, 5.00]	2.00 [1.00, 5.00]	2.00 [1.00, 5.00]
**Relationship status (N)**				
married	5583 (35.4%)	1608 (38.2%)	3950 (34.5%)	25 (17.6%)
widowed	229 (1.5%)	41 (1.0%)	188 (1.6%)	0 (0%)
divorced	707 (4.5%)	118 (2.8%)	587 (5.1%)	2 (1.4%)
separated	229 (1.5%)	62 (1.5%)	166 (1.5%)	1 (0.7%)
in a domestic partnership or civil union	566 (3.6%)	146 (3.5%)	416 (3.6%)	4 (2.8%)
in a steady relationship living together	2326 (14.7%)	519 (12.3%)	1798 (15.7%)	9 (6.3%)
in a steady relationship living apart	1811 (11.5%)	465 (11.0%)	1323 (11.6%)	23 (16.2%)
single	4158 (26.3%)	1205 (28.6%)	2890 (25.3%)	63 (44.4%)
other	181 (1.1%)	45 (1.1%)	121 (1.1%)	15 (10.6%)
**People in household (N)**				
1	3280 (20.8%)	884 (21.0%)	2359 (20.6%)	37 (26.1%)

2	5559 (35.2%)	1447 (34.4%)	4070 (35.6%)	42 (29.6%)
3-4	5681 (36.0%)	1540 (36.6%)	4090 (35.8%)	51 (35.9%)
5-6	1122 (7.1%)	297 (7.1%)	819 (7.2%)	6 (4.2%)
more than 6	148 (0.9%)	41 (1.0%)	101 (0.9%)	6 (4.2%)
**People underage (<18) in household (N)**				
no	11299 (71.6%)	3080 (73.2%)	8104 (70.8%)	115 (81.0%)
yes	4491 (28.4%)	1129 (26.8%)	3335 (29.2%)	27 (19.0%)
**Education (years)**				
Mean (SD)	18.4 (3.7)	18.4 (3.8)	18.5 (3.6)	18.0 (5.0)
0-12	1275 (8.1%)	372 (8.8%)	884 (7.7%)	19 (13.4%)
13-16	4889 (31.0%)	1349 (32.1%)	3490 (30.5%)	50 (35.2%)
17-20	7092 (44.9%)	1797 (42.7%)	5242 (45.8%)	53 (37.3%)
21 +	2534 (16.0%)	691 (16.4%)	1823 (15.9%)	20 (14.1%)
**Household income (€)**				
0-4.999	1446 (9.2%)	343 (8.1%)	1069 (9.3%)	34 (23.9%)
5.000-9.999	1311 (8.3%)	283 (6.7%)	999 (8.7%)	29 (20.4%)
10.000-14.999	1517 (9.6%)	358 (8.5%)	1139 (10.0%)	20 (14.1%)
15.000-24.999	2279 (14.4%)	602 (14.3%)	1661 (14.5%)	16 (11.3%)
25.000-49.999	3967 (25.1%)	1080 (25.7%)	2863 (25.0%)	24 (16.9%)
50.000-74.999	2357 (14.9%)	667 (15.8%)	1682 (14.7%)	8 (5.6%)
75.000-99.999	1285 (8.1%)	389 (9.2%)	890 (7.8%)	6 (4.2%)
100.000 +	1628 (10.3%)	487 (11.6%)	1136 (9.9%)	5 (3.5%)
**Country of residence (N)**				
Belgium	649 (4.1%)	146 (3.5%)	497 (4.3%)	6 (4.2%)
Germany	4803 (30.4%)	1248 (29.7%)	3505 (30.6%)	50 (35.2%)
Hong Kong	1073 (6.8%)	374 (8.9%)	695 (6.1%)	4 (2.8%)
Hungary	736 (4.7%)	177 (4.2%)	551 (4.8%)	8 (5.6%)
Italy	750 (4.7%)	292 (6.9%)	451 (3.9%)	7 (4.9%)
Netherlands	2051 (13.0%)	590 (14.0%)	1453 (12.7%)	8 (5.6%)
Poland	1715 (10.9%)	348 (8.3%)	1343 (11.7%)	24 (16.9%)
Serbia	543 (3.4%)	153 (3.6%)	390 (3.4%)	0 (0%)
Other	3470 (22.0%)	881 (20.9%)	2554 (22.3%)	35 (24.6%)
**Currently out of town (N)**				
yes	923 (5.8%)	219 (5.2%)	689 (6.0%)	15 (10.6%)
no	14867 (94.2%)	3990 (94.8%)	10750 (94.0%)	127 (89.4%)
**Nationality (N)**				
Belgium	588 (3.7%)	127 (3.0%)	456 (4.0%)	5 (3.5%)
Germany	4675 (29.6%)	1199 (28.5%)	3429 (30.0%)	47 (33.1%)
Hong Kong	1062 (6.7%)	367 (8.7%)	689 (6.0%)	6 (4.2%)
Hungary	811 (5.1%)	188 (4.5%)	613 (5.4%)	10 (7.0%)
Italy	851 (5.4%)	321 (7.6%)	519 (4.5%)	11 (7.7%)
Netherlands	1960 (12.4%)	573 (13.6%)	1381 (12.1%)	6 (4.2%)
Poland	1806 (11.4%)	368 (8.7%)	1415 (12.4%)	23 (16.2%)
Serbia	568 (3.6%)	164 (3.9%)	404 (3.5%)	0 (0%)
Other	3469 (22.0%)	902 (21.4%)	2533 (22.1%)	34 (23.9%)
**Survey language (N)**				
Arabic	18 (0.1%)	8 (0.2%)	10 (0.1%)	0 (0%)
Chinese	1186 (7.5%)	413 (9.8%)	767 (6.7%)	6 (4.2%)
Croatian	148 (0.9%)	24 (0.6%)	124 (1.1%)	0 (0%)
Czech	37 (0.2%)	11 (0.3%)	26 (0.2%)	0 (0%)
Danish	298 (1.9%)	75 (1.8%)	222 (1.9%)	1 (0.7%)
Dutch	2162 (13.7%)	598 (14.2%)	1555 (13.6%)	9 (6.3%)
English	1780 (11.3%)	575 (13.7%)	1168 (10.2%)	37 (26.1%)
Estonian	98 (0.6%)	16 (0.4%)	81 (0.7%)	1 (0.7%)
Finnish	18 (0.1%)	5 (0.1%)	13 (0.1%)	0 (0%)
French	375 (2.4%)	74 (1.8%)	297 (2.6%)	4 (2.8%)
German	4621 (29.3%)	1150 (27.3%)	3433 (30.0%)	38 (26.8%)
Greek	1 (0.0%)	0 (0%)	1 (0.0%)	0 (0%)

Hebrew	218 (1.4%)	48 (1.1%)	169 (1.5%)	1 (0.7%)
Hungarian	778 (4.9%)	182 (4.3%)	585 (5.1%)	11 (7.7%)
Italian	610 (3.9%)	220 (5.2%)	386 (3.4%)	4 (2.8%)
Lithuanian	3 (0.0%)	0 (0%)	3 (0.0%)	0 (0%)
Norwegian	226 (1.4%)	58 (1.4%)	167 (1.5%)	1 (0.7%)
Polish	1778 (11.3%)	364 (8.6%)	1391 (12.2%)	23 (16.2%)
Portuguese	130 (0.8%)	34 (0.8%)	96 (0.8%)	0 (0%)
Serbian	593 (3.8%)	174 (4.1%)	419 (3.7%)	0 (0%)
Slovak	426 (2.7%)	106 (2.5%)	314 (2.7%)	6 (4.2%)
Spanish	108 (0.7%)	45 (1.1%)	63 (0.6%)	0 (0%)
Swedish	165 (1.0%)	28 (0.7%)	137 (1.2%)	0 (0%)
Ukrainian	13 (0.1%)	1 (0.0%)	12 (0.1%)	0 (0%)
**Occupation (N)**				
Undergoing Education	2648 (16.8%)	662 (15.7%)	1931 (16.9%)	55 (38.7%)
Education or research	3901 (24.7%)	935 (22.2%)	2932 (25.6%)	34 (23.9%)
Arts, entertainment, sports and media	1263 (8.0%)	360 (8.6%)	878 (7.7%)	25 (17.6%)
Healthcare	2496 (15.8%)	443 (10.5%)	2040 (17.8%)	13 (9.2%)
First responder (paramedic/firefighter/police)	152 (1.0%)	64 (1.5%)	88 (0.8%)	0 (0%)
Military	45 (0.3%)	30 (0.7%)	14 (0.1%)	1 (0.7%)
Civil services, politics	920 (5.8%)	241 (5.7%)	669 (5.8%)	10 (7.0%)
Finance and economy	848 (5.4%)	280 (6.7%)	567 (5.0%)	1 (0.7%)
Industry	556 (3.5%)	305 (7.2%)	251 (2.2%)	0 (0%)
Sales and services (incl. restaurants and bars)	1016 (6.4%)	302 (7.2%)	702 (6.1%)	12 (8.5%)
Transport (goods and people)	203 (1.3%)	104 (2.5%)	98 (0.9%)	1 (0.7%)
Installation, maintenance, cleaning, and repairs	165 (1.0%)	129 (3.1%)	36 (0.3%)	0 (0%)
Construction	165 (1.0%)	93 (2.2%)	72 (0.6%)	0 (0%)
Farming, fishing, and forestry	98 (0.6%)	39 (0.9%)	58 (0.5%)	1 (0.7%)
Office and administrative support	2078 (13.2%)	490 (11.6%)	1581 (13.8%)	7 (4.9%)
(Currently) not working	1669 (10.6%)	469 (11.1%)	1179 (10.3%)	21 (14.8%)
Other	1974 (12.5%)	603 (14.3%)	1355 (11.8%)	16 (11.3%)
**Occupational status (N)**				
Temporary contract, full-time	1158 (7.3%)	325 (7.7%)	825 (7.2%)	8 (5.6%)
Permanent contract, full-time	5761 (36.5%)	1853 (44.0%)	3886 (34.0%)	22 (15.5%)
Permanent contract, part-time	2097 (13.3%)	267 (6.3%)	1815 (15.9%)	15 (10.6%)
Temporary contract, part-time	778 (4.9%)	154 (3.7%)	614 (5.4%)	10 (7.0%)
Self-employed	1315 (8.3%)	427 (10.1%)	879 (7.7%)	9 (6.3%)
Working as a freelancer	864 (5.5%)	230 (5.5%)	617 (5.4%)	17 (12.0%)
On parental leave	272 (1.7%)	17 (0.4%)	253 (2.2%)	2 (1.4%)
On sick leave (long term)	221 (1.4%)	34 (0.8%)	186 (1.6%)	1 (0.7%)
Unemployed on social benefit	242 (1.5%)	80 (1.9%)	157 (1.4%)	5 (3.5%)
Unemployed without social benefit	519 (3.3%)	129 (3.1%)	377 (3.3%)	13 (9.2%)
Full-time studying	2436 (15.4%)	604 (14.4%)	1781 (15.6%)	51 (35.9%)
Retired	1109 (7.0%)	393 (9.3%)	711 (6.2%)	5 (3.5%)
Other	1049 (6.6%)	192 (4.6%)	843 (7.4%)	14 (9.9%)

Table S1. Sample characteristics (N=15,790 complete valid data sets). Note: Past or present diagnosed mental health condition: question was only asked after 406 participants had

provided valid and complete data sets (512 complete but partially invalid data sets from European residents; see Suppl. 1). Household income: ranges were adapted after 355 participants (of whom 346 European residents) had provided complete data sets (see Suppl. 1); those early participants were removed from the analysis as invalid during data cleaning and are not reported here. Percentages refer to the whole valid sample of 15,790 participants with complete data. For details on diagnoses, see Table S4. Good general health: frequency of falling ill compared to others (1 = more frequently ill; 5 = never ill). Number of underage people in household was dichotomized for simplicity. Years of education: sum of years spent in any form of education, including elementary school and professional higher education. Household income: average annual household income, asked to be estimated in Euro. Depending on the language the survey questionnaire was completed in, income ranges were converted to the currency of the country associated with that language (e.g., Shekel, Forint and Złoty for Hebrew, Hungarian and Polish questionnaires, respectively. Occupation and occupational status: numbers do not add up to 15,790 / 100% since multiple answers were possible. Not shown are: if tested positive for COVID-19: severity of symptoms; city/town of residence; if out of town: current geographical location.

**Questionnaire element**	**Source of questions**
Basic socio-demographic and health information	self-generated
Mental health	GHQ-12 (Goldberg et al., 1997)
Perceived social support	SOZU-K-10 (Dunkel et al., 2005)*
Perceived change in social support during the Corona crisis	self-generated
Optimism	self-generated
Perceived general self-efficacy	English version of ASKU (Beierlein et al., 2012)
Perceived good stress recovery	BRS (Smith et al., 2008)
Neuroticism	selection from BFI-10 (Rammstedt and John, 2007; see 2.2.2)
Positive appraisal style	PASS (selection from brief COPE (Carver, 1997) and CERQ-short (Garnefski and Kraaij, 2006) and self-generated; see detailed explanation in 2.2.2)
Behavioral coping style	BCS (selection from brief COPE (Carver, 1997); see 2.2.2)
Positive appraisal specifically of the Corona crisis	self-generated
Corona pandemic-specific stressor exposure	self-generated (see 2.3.2 and Table S6)
General stressor exposure	self-generated (see 2.3.2 and Table S6)
Open questions for exploratory search strategy	self-generated

*available only in German, from there translated into English

Table S2. Questionnaire elements: psychological constructs and used instruments. Note: existing instruments have partly been shortened or adapted.

Variable/index	Used questionnaire element(s)
Mental health problems (P)	Mental health (sum score, item scoring 0 to 3)
Perceived social support (PSS)	Perceived social support (sum score, item scoring 1 to 5)
Perceived change in social support during the Corona crisis (CSS)	Perceived change in social support during the Corona crisis (item scoring 1 to 5)
Optimism (OPT)	Optimism (item scoring 1 to 7)
Perceived general self-efficacy (GSE)	Perceived general self-efficacy (sum score, item scoring 1-5)
Perceived good stress recovery (REC)	Perceived good stress recovery (mean score, item scoring 1 to 5)
Neuroticism (NEU)	Neuroticism (sum score, item scoring -2 to 2)
Positive appraisal style (PAS)	Positive appraisal style (composite score, taking the average of the z-normalized scores of the COPE items (item scoring 1 to 4), the CERQ items (item scoring 1 to 5), and the self-generated items (item scoring 1 to 5)).
Behavioral coping style (BCS)	Behavioral coping style (sum score, item scoring 1 to 4)
Positive appraisal specifically of the Corona crisis (PAC)	Positive appraisal specifically of the Corona crisis (sum score, item scoring 1 to 5)
Corona pandemic-specific stressor exposure (E_S)	Corona pandemic-specific stressor exposure (see 2.3.2 and 2.3.3 for details)
General stressor exposure (E_G)	General stressor exposure (see 2.3.2 and 2.3.3 for details)
Resilience (RES)	Mental health, Corona pandemic-specific stressor exposure, General stressor exposure (see 2.3.4 for details)

Table S3. Variables, indices and covariates. Note: covariates are calculated based on the basic socio-demographic and health information provided in the survey questionnaire (see also Table S1). Items are age, gender, nationality, country of residence, city/town of residence, current stay out of town for longer period, if so: current geographical location, years of education, occupation, occupational status, average annual household income, relationship status, people living in household, of those under the age of 18, general health status, past or present diagnosed mental health condition (only asked starting with complete and valid respondent 407; see also Suppl. 1), if so: details (open answer), belonging to COVID-19 risk group, COVID-19 infection test status, if positive: date of diagnosis and severity of symptoms, quarantine status, opinion about authorities' measures to curtail virus spread, adherence to recommended procedures to limit virus spread.

Covariates not statistically tested for association with resilience were: city/town of residence, if current stay out of town for longer period: current geographical location, if past or present diagnosed mental health condition: details (open answer), if positive COVID-19 infection test: date of diagnosis and symptom severity.

Among the covariates tested using separate univariate regression analyses, the following survived a likelihood ratio test at $p<0.2$ and were included in all further analyses: gender, age, country of residence, household income, years of education, occupation (grouping: student or employee working in research/education vs. not), occupational status (permanent contract vs. not), relationship status, people in household, underage people in household, general health status, and opinion about authorities' measures to curtail virus spread. Grouping of covariates was as described in the preregistration amendment (osf.io/thka9), with the exception of Serbia as a country of residence level that had erroneously been missed when calculating subgroup sizes for the amendment and was thus added here. Past or present diagnosed mental health condition was added in separate analyses of the subsample that was interrogated about mental health.

**Diagnosis**	**Count**
Unknown/unclear	90
Burnout	142
Mental and behavioural disorders due to psychoactive substance use (F10–F19)	19
Schizophrenia, schizotypal and delusional disorders (F20–F29)	35
Manic episode/bipolar affective disorder (F30–F31)	122
Depressive episode/recurrent depressive disorder (F32–F33)	2019
Persistent mood (affective) disorders (F34)	41
Phobic/other anxiety disorders (F40–F41)	1025
Obsessive-compulsive disorder (F42)	73
Post-traumatic stress disorder (F43.1)	284
Other neurotic, stress-related and somatoform disorders (F40–F48)	251
Behavioural syndromes associated with physiological disturbances and physical factors (F50–F59)	161
Borderline personality disorder (F60.3)	112
Other disorders of adult personality and behaviour (F60–F69)	95
Pervasive developmental disorders (F84)	83
Behavioural and emotional disorders with onset usually occurring in childhood and adolescence (F90–F98)	113
**Total**	**4665**

Table S4. Past or present diagnosed mental health conditions. Note: based on n=15,384 completed surveys, valid after data cleaning. The diagnosis question was only introduced into the questionnaire after n=406 participants had provided complete and valid data (and 512 European residents had provided complete but partially invalid data, see Suppl. 1.) n=3607 participants affirmed the question. Diagnostic categorization (F indices) was done following ICD-10 based on participants' free-text answers by an experienced psychiatrist and an experienced clinical psychologist. Burnout was added as an extra category. n=91 affirmative answers could not be considered a diagnosis. Of the remaining n=3516 participants (22.9%), several indicated multiple diagnoses.

**Primary hypothesis**	
H1	Positive appraisal style (PAS) is positively associated with resilience
**Key secondary hypotheses**	
H2	Perceived social support (PSS) is positively associated with resilience
H3	A perceived increase in social support during the Corona crisis (CSS) is positively associated with resilience
H4	Optimism (OPT) is positively associated with resilience
H5	Perceived general self-efficacy (GSE) is positively associated with resilience
H6	Perceived good stress recovery (REC) is positively associated with resilience
H7	Neuroticism (NEU) is negatively associated with resilience
H8	Behavioral coping style (BCS) is positively associated with resilience
H9	Positive appraisal specifically of the Corona crisis (PAC) is positively associated with resilience
**Secondary hypotheses**	
H10	The expected positive effect of social support (PSS) on resilience is positively mediated by its effect on positive appraisal style (PAS)
H11	The expected positive effect of positive appraisal style (PAS) on resilience is positively mediated by its effect on perceived good stress recovery (REC)

Table S5. A priori hypotheses. Note: see preregistration at osf.io/r6btn.

Stressors	Count (out of 15970)	Frequency (%)	Aver. severity (1-5)
**General stressors (for calculation of E_G)**			
Negative political events.	13152	83.29	3.13
Conflicts or disagreements in family, social, or professional settings.	9755	61.78	2.91
Financial problems.	7343	46.5	2.96
Myself or a close person experienced physical health problems.	7797	49.38	3.17
Myself or a close person experienced mental health problems.	7598	48.12	3.29
Burdensome experiences at home or with your family (e.g., caring for/looking after relatives).	6771	42.88	2.89
Burdensome experiences at work, school, university, or another occupation.	9704	61.46	2.98
Burdensome environmental experiences (e.g., pollution, noise, unsafe neighborhood, ...).	6773	42.89	2.49
Conflicts with strangers (e.g., authorities, criminals).	3098	19.62	2.62
Separation from a loved one.	7121	45.1	3.56
Death of a loved one.	1684	10.66	3.85
other	1937	12.27	3.8
**Corona crisis-specific stressors (for calculation of E_S)**			
Having COVID-19 symptoms, or symptoms that could be related to COVID-19.	6411	40.60	2.83
COVID-19 symptoms, or symptoms that could be related to COVID-19 in family members, friends, loved ones, or colleagues.	7664	48.54	3.08
Being at increased risk for an infection (e.g., at work).	9504	60.19	2.85
Being at increased risk for a serious course of the disease in case of an infection (belonging to a so-called 'risk group').	6250	39.58	2.99
Family, friends, or loved ones being at increased risk for a serious course of the disease in case of an infection (they belong to a so-called 'risk group').	13090	82.90	3.50
Problems with access to healthcare, medication, or sanitation.	7447	47.16	2.96
You cannot return to the country you live in.	1734	10.98	3.32
(Feeling) restricted to leave your home.	13531	85.69	3.01
Loss of social contact.	13895	88.00	3.18
Unable to attend an important social event.	12937	81.93	2.95
Family, friends, or loved ones are at the hospital and you are restricted in visiting them.	2871	18.18	3.66
Unable to attend a funeral of a family member, friend, or loved one.	2225	14.09	3.75
Family, friends, or loved ones serving in the army or as first responder (paramedic/firefighter/police).	3789	24.00	2.97
Family, friends, or loved ones working as health care professional.	7986	50.58	2.9
Not being able to perform leisure activities.	14215	90.03	3.07
Less physical activity than usual.	12613	79.88	3.14
Problems arranging childcare.	3102	19.65	3.25

Difficulties combining work with childcare.	3203	20.28	3.4
Tensions at home or family conflict	8383	53.09	2.78
Private travel not possible.	12235	77.49	3.08
Being at an increased risk for economic damage in your occupation.	8200	51.93	3.20
Increased work load.	7491	47.44	2.94
Work-related delays/obstacles.	10133	64.17	3.10
Business travel not possible.	4772	30.22	2.59
(Threat of) job loss or insolvency of private company.	4507	28.54	3.38
(Threat of) job loss or insolvency of private company for someone in your household.	5032	31.87	3.31
Problems obtaining basic needs.	8397	53.18	2.59
Problems obtaining other goods and services.	10056	63.69	2.48
Corona-related media coverage	14646	92.75	3.20
other	4098	25.95	3.95

Table S6. Assessed stressors, stressor counts and average severity ratings.

	PAS	PSS	CSS	OPT	GSE	REC	NEU	BCS	PAC	RES_C
**PAS**	**1.00**	0.28	0.14	0.44	0.41	0.44	-0.37	0.27	0.37	0.29
**PSS**	0.28	**1.00**	0.26	0.35	0.29	0.30	-0.24	0.50	0.25	0.25
**CSS**	0.14	0.26	**1.00**	0.13	0.12	0.12	-0.09	0.16	0.18	0.16
**OPT**	0.44	0.35	0.13	**1.00**	0.45	0.52	-0.50	0.15	0.30	0.36
**GSE**	0.41	0.29	0.12	0.45	**1.00**	0.54	-0.47	0.11	0.19	0.31
**REC**	0.44	0.30	0.12	0.52	0.54	**1.00**	-0.63	0.05	0.22	0.39
**NEU**	-0.37	-0.24	-0.09	-0.50	-0.47	-0.63	**1.00**	0.03	-0.17	-0.36
**BCS**	0.27	0.50	0.16	0.15	0.11	0.05	0.03	**1.00**	0.18	0.09
**PAC**	0.37	0.25	0.18	0.30	0.19	0.22	-0.17	0.18	**1.00**	0.27
**RES_C**	0.29	0.25	0.16	0.36	0.31	0.39	-0.36	0.09	0.27	**1.00**

Table S7. Correlations of independent variables (hypothesized resilience factors) and outcome-based resilience (RES_C). Note: PAS, positive appraisal style; PSS, perceived social support; CSS, increase in perceived social support during the Corona crisis; OPT, optimism; GSE, perceived general self-efficacy; REC, perceived good stress recovery; NEU, neuroticism; BCS, behavioral coping style; PAC, positive appraisal specifically of the Corona crisis; RES_C, resilience to all stressors combined (main outcome)

Table S8: Tests of directed hypotheses H1-H9 (outcome: resilience to all stressors combined, RES_C)

	DV: RES_C (E_C; SSM)								
PAS	.26 (.24;.28)***								
PSS		.23 (.21;.25)***							
CSS			.13 (.11;.15)***						
OPT				.30 (.28;.32)***					
GSE					.26 (.24;.28)***				
REC						.34 (.32;.36)***			
NEU							-.31 (-.33;-.29)***		
BCS								.11 (.09;.13)***	
PAC									.24 (.22;.26)***
Gender: Female[a]	-.11 (-.15;-.06)***	-.16 (-.20;-.11)***	-.10 (-.15;-.06)***	-.11 (-.15;-.06)***	-.08 (-.13;-.04)***	-.04 (-.08;.01)	.002 (-.04;.05)	-.14 (-.18;-.09)***	-.12 (-.16;-.07)***
Gender: Diverse[a]	-.21 (-.41;.0001)	-.23 (-.44;-.02)*	-.21 (-.42;-.002)*	-.16 (-.37;.04)	-.13 (-.34;.08)	-.05 (-.26;.15)	-.08 (-.29;.12)	-.24 (-.45;-.03)*	-.21 (-.42;-.01)*
Age: 31-45	.10 (.04;.15)***	.14 (.08;.20)***	.10 (.04;.15)***	.08 (.02;.14)**	.06 (.01;.12)*	.07 (.02;.13)**	.06 (.005;.12)*	.10 (.05;.16)***	.08 (.03;.14)**
Age: 46-60	.21 (.15;.28)***	.27 (.20;.33)***	.21 (.14;.27)***	.16 (.09;.22)***	.17 (.10;.23)***	.15 (.09;.22)***	.12 (.06;.18)***	.23 (.17;.30)***	.20 (.13;.26)***
Age: 61+	.29 (.20;.37)***	.34 (.25;.42)***	.25 (.17;.34)***	.21 (.12;.29)***	.22 (.14;.31)***	.17 (.09;.26)***	.17 (.08;.25)***	.31 (.23;.40)***	.29 (.20;.37)***
Country of residence: Belgium[a]	-.15 (-.25;-.05)***	-.16 (-.26;-.06)***	-.16 (-.26;-.05)***	-.12 (-.22;-.02)*	-.12 (-.22;-.02)*	-.12 (-.22;-.02)*	-.13 (-.22;-.03)*	-.15 (-.25;-.05)**	-.22 (-.32;-.12)***
Country of residence: Hong Kong[a]	-.14 (-.24;-.04)**	-.08 (-.18;.02)	-.12 (-.22;-.01)*	-.01 (-.11;.09)	.08 (-.02;.18)	.01 (-.09;.11)	-.04 (-.14;.06)	-.13 (-.23;-.03)*	-.19 (-.29;-.09)***
Country of residence: Hungary[a]	-.34 (-.44;-.24)***	-.38 (-.48;-.28)***	-.38 (-.48;-.28)***	-.36 (-.46;-.26)***	-.43 (-.53;-.33)***	-.32 (-.42;-.22)***	-.35 (-.44;-.25)***	-.43 (-.53;-.32)***	-.39 (-.49;-.29)***
Country of residence: Italy[a]	-.17 (-.27;-.08)***	-.11 (-.21;-.01)*	-.19 (-.29;-.08)***	-.14 (-.24;-.05)**	-.18 (-.28;-.08)***	-.16 (-.26;-.06)***	-.18 (-.28;-.08)***	-.19 (-.29;-.09)***	-.25 (-.35;-.15)***
Country of residence: Netherlands[a]	-.37 (-.44;-.31)***	-.38 (-.45;-.32)***	-.36 (-.42;-.29)***	-.36 (-.42;-.29)***	-.35 (-.42;-.29)***	-.31 (-.38;-.25)***	-.37 (-.43;-.31)***	-.37 (-.44;-.31)***	-.39 (-.45;-.32)***
Country of residence: Poland[a]	-.15 (-.22;-.07)***	-.12 (-.20;-.05)***	-.21 (-.29;-.14)***	-.11 (-.19;-.04)***	-.17 (-.24;-.09)***	-.10 (-.17;-.03)**	-.08 (-.15;-.01)*	-.20 (-.28;-.13)***	-.15 (-.23;-.08)***
Country of residence: Serbia[a]	.25 (.13;.36)***	.27 (.15;.38)***	.26 (.14;.38)***	.26 (.14;.37)***	.22 (.10;.33)***	.25 (.13;.36)***	.29 (.17;.40)***	.30 (.18;.42)***	.24 (.13;.36)***
Country of residence: Other[a]	-.04 (-.10;.01)	.01 (-.04;.07)	-.03 (-.09;.03)	-.03 (-.08;.02)	-.02 (-.08;.03)	-.03 (-.08;.02)	-.02 (-.07;.03)	-.02 (-.08;.04)	-.08 (-.13;-.02)**
Household income: €5,000-€9,999[a]	-.003 (-.10;.09)	-.01 (-.10;.08)	-.02 (-.12;.07)	.01 (-.08;.10)	-.02 (-.11;.08)	-.01 (-.10;.08)	-.02 (-.11;.07)	-.02 (-.11;.08)	.01 (-.08;.10)
Household income: €10,000-€14,999[a]	-.004 (-.09;.08)	-.03 (-.12;.06)	-.02 (-.12;.07)	-.02 (-.11;.07)	-.04 (-.13;.05)	-.03 (-.11;.06)	-.02 (-.11;.07)	-.02 (-.11;.07)	.01 (-.08;.10)
Household income: €15,000-€24,999[a]	.05 (-.03;.13)	.02 (-.06;.10)	.03 (-.05;.12)	.03 (-.05;.11)	.01 (-.07;.09)	.03 (-.05;.11)	.03 (-.05;.11)	.03 (-.05;.12)	.06 (-.02;.14)
Household income: €25,000-€49,999[a]	.06 (-.02;.13)	.02 (-.06;.09)	.05 (-.03;.12)	.04 (-.04;.11)	.004 (-.07;.08)	.01 (-.06;.09)	.03 (-.04;.11)	.05 (-.03;.13)	.07 (-.01;.14)
Household income: €50,000-€74,999[a]	.06 (-.02;.15)	.03 (-.06;.11)	.05 (-.03;.14)	.04 (-.04;.13)	.004 (-.08;.09)	.01 (-.07;.09)	.02 (-.06;.10)	.06 (-.03;.15)	.07 (-.01;.16)
Household income: €75,000-€99,999[a]	.05 (-.05;.14)	.002 (-.10;.10)	.05 (-.05;.15)	.02 (-.08;.12)	-.01 (-.11;.09)	-.02 (-.11;.08)	.01 (-.09;.11)	.05 (-.05;.15)	.07 (-.03;.17)
Household income: €10,000+[a]	.05 (-.04;.14)	.01 (-.08;.11)	.06 (-.04;.15)	.04 (-.05;.13)	-.02 (-.11;.08)	-.02 (-.11;.07)	.01 (-.08;.10)	.06 (-.04;.16)	.07 (-.02;.17)
Education: 13-16yrs[a]	.11 (.03;.19)**	.12 (.04;.19)***	.13 (.05;.21)***	.12 (.04;.19)***	.11 (.03;.19)**	.13 (.05;.20)***	.12 (.04;.19)***	.13 (.05;.21)***	.13 (.05;.20)***
Education: 17-20yrs[a]	.14 (.06;.21)***	.15 (.07;.22)***	.17 (.10;.25)***	.15 (.07;.22)***	.13 (.05;.20)***	.15 (.08;.22)***	.15 (.08;.22)***	.16 (.08;.23)***	.15 (.08;.23)***
Education: 21+ yrs[a]	.19 (.11;.28)***	.21 (.13;.30)***	.23 (.14;.32)***	.21 (.12;.29)***	.19 (.10;.27)***	.21 (.12;.29)***	.21 (.13;.30)***	.22 (.14;.31)***	.22 (.13;.30)***
Occupation: Student/ working in research/education[a]	-.003 (-.05;.04)	-.01 (-.06;.03)	.01 (-.04;.05)	-.01 (-.05;.04)	-.01 (-.06;.03)	-.003 (-.05;.04)	.003 (-.04;.05)	.001 (-.04;.05)	.003 (-.04;.05)
Occupational status: Permanent contract[a]	.08 (.04;.13)***	.07 (.03;.12)***	.08 (.04;.12)***	.06 (.02;.10)**	.06 (.02;.11)***	.07 (.02;.11)***	.06 (.02;.10)**	.09 (.04;.13)***	.08 (.04;.13)***
Relationship status: Widowed[a]	.11 (.05;.17)***	.04 (-.02;.10)	.11 (.05;.18)***	.08 (.02;.14)**	.11 (.05;.17)***	.07 (.01;.13)*	.11 (.05;.17)***	.10 (.03;.16)***	.11 (.05;.17)***
Relationship status: Divorced[a]	-.05 (-.22;.12)	-.11 (-.28;.06)	-.06 (-.23;.11)	-.10 (-.27;.06)	-.07 (-.24;.10)	-.08 (-.25;.08)	-.07 (-.24;.09)	-.08 (-.25;.10)	-.04 (-.21;.13)

Relationship status: Separated[a]	.04 (-.07;.14)	.04 (-.06;.14)	.07 (-.04;.17)	.003 (-.10;.10)	.01 (-.09;.11)	.001 (-.10;.10)	.03 (-.08;.13)	.05 (-.06;.15)	.04 (-.06;.14)
Relationship status: Domestic	.04 (-.13;.20)	.03 (-.13;.20)	.03 (-.13;.20)	.005 (-.16;.17)	.03 (-.14;.19)	.02 (-.14;.18)	.01 (-.15;.17)	.02 (-.15;.19)	.03 (-.14;.19)
Relationship status: Steady relationship, living together[a]	.15 (.04;.26)**	.06 (-.05;.17)	.14 (.02;.25)*	.13 (.02;.23)*	.13 (.02;.24)*	.13 (.02;.24)*	.16 (.05;.26)**	.12 (.004;.23)*	.16 (.05;.28)**
Relationship status: Steady relationship, living apart[a]	.14 (.08;.21)***	.07 (.01;.14)*	.15 (.08;.21)***	.12 (.06;.19)***	.14 (.08;.20)***	.11 (.05;.17)***	.14 (.07;.20)***	.13 (.07;.20)***	.15 (.08;.21)***
Relationship status: Single[a]	.04 (-.02;.11)	-.03 (-.10;.04)	.04 (-.03;.11)	.03 (-.04;.09)	.04 (-.03;.10)	.04 (-.03;.10)	.05 (-.02;.12)	.02 (-.05;.09)	.04 (-.03;.10)
Relationship status: Other[a]	.10 (-.09;.28)	.09 (-.09;.28)	.11 (-.07;.30)	.10 (-.08;.28)	.10 (-.09;.28)	.08 (-.10;.26)	.08 (-.10;.26)	.10 (-.08;.29)	.09 (-.09;.27)
People in household	.03 (.005;.05)*	.03 (.002;.05)*	.04 (.02;.07)***	.03 (.01;.06)**	.04 (.02;.07)***	.03 (.01;.06)**	.04 (.01;.06)***	.03 (.01;.06)**	.02 (-.001;.05)
People underage (<18) in household	.01 (-.001;.03)	.02 (.003;.03)*	.01 (.001;.03)*	.01 (-.004;.02)	.01 (.0004;.03)*	.01 (-.001;.03)	.01 (.001;.03)*	.01 (-.0004;.03)	.01 (.001;.03)*
General health status	.08 (.06;.10)***	.10 (.08;.12)***	.12 (.10;.14)***	.06 (.04;.08)***	.07 (.05;.09)***	.03 (.01;.05)***	.05 (.03;.07)***	.12 (.10;.14)***	.10 (.08;.12)***
Opinion about authorities' measures	.06 (.04;.08)***	.06 (.04;.08)***	.07 (.05;.09)***	.05 (.03;.07)***	.07 (.05;.09)***	.07 (.05;.09)***	.07 (.05;.09)***	.08 (.06;.10)***	.03 (.01;.05)***
Constant	-.72 (-.87;-.56)***	-.68 (-.84;-.52)***	-.91 (-1.07;-.76)***	-.58 (-.74;-.43)***	-.69 (-.85;-.54)***	-.61 (-.76;-.46)***	-.74 (-.89;-.58)***	-.89 (-1.05;-.73)***	-.65 (-.80;-.49)***
Adjusted R^2 increase by RF	0.06	0.05	0.02	0.08	0.06	0.09	0.08	0.01	0.05
Observations	15,790	15,790	15,790	15,790	15,790	15,790	15,790	15,790	15,790
R^2	.15	.14	.11	.17	.15	.18	.17	.10	.14
Adjusted R^2	.15	.13	.10	.17	.14	.18	.17	.10	.14
Residual Std. Error (df = 15751)	.92	.93	.95	.91	.93	.90	.91	.95	.93
F Statistic (df = 38; 15751)	73.14***	65.22***	49.11***	83.46***	70.61***	92.88***	83.84***	45.72***	68.60***

Note: $^{*}p<0.01$; $^{**}p<0.001$; $^{***}p<0.0001$

99% CI in parentheses

[a]Reference categories: Male (*Gender*), 18-30 (*Age*), Germany (*Country of residence*), €0-€4,999 (*Household income*), <12 yrs (*Education*), Not a student / working in research/education (*Occupation*), No permanent contract (*Occupational status*), Married (*Relationship status*)

Table S8: Tests of directed hypotheses H1-H9 (outcome: resilience to all stressors combined, RES_C). Note: a multiple regression was calculated separately for each hypothesized resilience factor.

Table S9: Tests of directed hypotheses H1-H9 (outcome: resilience to general stressors, RES_G)

	DV: *RES_G (E_G; SSM)*								
PAS	.24 (.22;.26)***								
PSS		.22 (.20;.24)***							
CSS			.13 (.11;.15)***						
OPT				.29 (.27;.31)***					
GSE					.24 (.22;.26)***				
REC						.33 (.31;.35)***			
NEU							-.30 (-.32;-.28)***		
BCS								.09 (.07;.11)***	
PAC									.24 (.22;.26)***
Gender: Female[a]	-.10 (-.15;-.06)***	-.15 (-.20;-.11)***	-.10 (-.15;-.06)***	-.11 (-.15;-.06)***	-.08 (-.13;-.04)***	-.04 (-.08;.005)	.001 (-.04;.04)	-.13 (-.17;-.08)***	-.12 (-.16;-.07)***
Gender: Diverse[a]	-.12 (-.33;.09)	-.15 (-.35;.06)	-.13 (-.34;.08)	-.08 (-.29;.12)	-.05 (-.26;.16)	.02 (-.18;.23)	-.003 (-.21;.20)	-.15 (-.36;.06)	-.13 (-.34;.08)
Age: 31-45	.06 (.001;.12)*	.10 (.04;.16)***	.06 (.001;.12)*	.04 (-.01;.10)	.03 (-.03;.09)	.04 (-.02;.09)	.03 (-.03;.08)	.07 (.01;.12)*	.05 (-.01;.10)
Age: 46-60	.22 (.16;.28)***	.27 (.21;.34)***	.21 (.15;.28)***	.17 (.10;.23)***	.18 (.11;.24)***	.16 (.10;.23)***	.13 (.07;.20)***	.23 (.17;.30)***	.21 (.14;.27)***
Age: 61+	.32 (.23;.40)***	.36 (.27;.45)***	.28 (.20;.37)***	.24 (.15;.32)***	.25 (.17;.34)***	.21 (.12;.29)***	.20 (.11;.28)***	.34 (.25;.42)***	.31 (.23;.40)***
Country of residence: Belgium[a]	-.18 (-.29;-.08)***	-.19 (-.29;-.09)***	-.19 (-.29;-.08)***	-.16 (-.26;-.05)***	-.16 (-.26;-.05)***	-.15 (-.25;-.05)**	-.16 (-.26;-.06)***	-.18 (-.29;-.08)***	-.25 (-.35;-.15)***
Country of residence: Hong Kong[a]	.03 (-.07;.13)	.08 (-.02;.18)	.05 (-.05;.15)	.15 (.05;.25)***	.23 (.13;.33)***	.17 (.07;.27)***	.12 (.02;.22)*	.04 (-.06;.14)	-.03 (-.13;.07)
Country of residence: Hungary[a]	-.24 (-.34;-.14)***	-.28 (-.38;-.18)***	-.28 (-.38;-.18)***	-.26 (-.36;-.16)***	-.32 (-.43;-.22)***	-.22 (-.32;-.12)***	-.25 (-.35;-.15)***	-.32 (-.42;-.22)***	-.29 (-.39;-.19)***
Country of residence: Italy[a]	-.19 (-.29;-.09)***	-.12 (-.22;-.02)*	-.20 (-.30;-.10)***	-.16 (-.26;-.06)***	-.19 (-.29;-.09)***	-.17 (-.27;-.08)***	-.19 (-.29;-.09)***	-.21 (-.31;-.10)***	-.26 (-.36;-.16)***
Country of residence: Other[a]	-.03 (-.09;.02)	.02 (-.04;.07)	-.02 (-.08;.03)	-.02 (-.08;.03)	-.01 (-.07;.04)	-.02 (-.08;.03)	-.01 (-.07;.04)	-.01 (-.07;.04)	-.07 (-.13;-.02)**
Country of residence: Netherlands[a]	-.42 (-.48;-.35)***	-.43 (-.49;-.36)***	-.41 (-.47;-.34)***	-.40 (-.47;-.34)***	-.40 (-.47;-.34)***	-.36 (-.43;-.30)***	-.42 (-.48;-.35)***	-.42 (-.49;-.36)***	-.43 (-.50;-.37)***
Country of residence: Poland[a]	-.17 (-.24;-.09)***	-.15 (-.22;-.07)***	-.23 (-.31;-.16)***	-.14 (-.21;-.06)***	-.19 (-.26;-.12)***	-.12 (-.19;-.05)***	-.10 (-.18;-.03)**	-.22 (-.30;-.14)***	-.17 (-.25;-.10)***
Country of residence: Serbia[a]	.29 (.17;.41)***	.31 (.19;.43)***	.30 (.18;.42)***	.30 (.19;.42)***	.27 (.15;.38)***	.29 (.18;.41)***	.33 (.21;.44)***	.34 (.22;.46)***	.29 (.17;.40)***
Household income: €5,000-€9,999[a]	-.02 (-.11;.08)	-.02 (-.12;.07)	-.03 (-.13;.06)	-.003 (-.09;.09)	-.03 (-.12;.06)	-.02 (-.12;.07)	-.03 (-.12;.06)	-.03 (-.12;.07)	-.003 (-.10;.09)
Household income: €10,000-€14,999[a]	-.02 (-.11;.07)	-.04 (-.13;.05)	-.04 (-.13;.05)	-.04 (-.13;.05)	-.06 (-.15;.03)	-.04 (-.13;.05)	-.04 (-.13;.05)	-.04 (-.13;.05)	-.01 (-.10;.08)
Household income: €15,000-€24,999[a]	.04 (-.04;.12)	.01 (-.07;.10)	.03 (-.06;.11)	.02 (-.06;.11)	.005 (-.08;.09)	.03 (-.05;.11)	.02 (-.06;.11)	.03 (-.06;.11)	.06 (-.03;.14)
Household income: €25,000-€49,999[a]	.02 (-.06;.09)	-.02 (-.10;.06)	.01 (-.07;.09)	-.003 (-.08;.07)	-.03 (-.11;.05)	-.03 (-.10;.05)	-.004 (-.08;.07)	.01 (-.07;.09)	.03 (-.05;.11)
Household income: €50,000-€74,999[a]	.02 (-.07;.10)	-.02 (-.10;.07)	.01 (-.08;.10)	-.003 (-.09;.08)	-.04 (-.12;.05)	-.04 (-.12;.05)	-.02 (-.11;.06)	.01 (-.07;.10)	.02 (-.06;.11)
Household income: €75,000-€99,999[a]	-.01 (-.11;.09)	-.05 (-.15;.05)	-.01 (-.11;.09)	-.03 (-.13;.06)	-.06 (-.16;.04)	-.07 (-.16;.03)	-.04 (-.14;.05)	-.002 (-.10;.10)	.01 (-.08;.11)
Household income: €10,000+[a]	-.02 (-.12;.07)	-.06 (-.15;.04)	-.01 (-.11;.08)	-.04 (-.13;.06)	-.09 (-.18;.01)	-.09 (-.19;-.002)*	-.06 (-.16;.03)	-.01 (-.11;.08)	-.002 (-.10;.09)
Education: 13-16yrs[a]	.08 (.01;.16)*	.09 (.01;.17)*	.10 (.03;.18)**	.09 (.02;.17)*	.08 (.01;.16)*	.10 (.02;.17)**	.09 (.01;.17)*	.10 (.02;.18)**	.10 (.02;.17)**
Education: 17-20yrs[a]	.09 (.01;.16)*	.10 (.02;.17)**	.12 (.05;.20)***	.10 (.02;.17)**	.08 (.01;.16)*	.10 (.03;.18)**	.10 (.03;.18)**	.11 (.03;.19)**	.10 (.03;.18)**
Education: 21+ yrs[a]	.13 (.05;.22)***	.15 (.06;.24)***	.17 (.08;.26)***	.15 (.06;.23)***	.12 (.04;.21)**	.14 (.06;.23)***	.15 (.06;.23)***	.16 (.07;.25)***	.15 (.06;.24)***
Occupation: Student / working in research/education[a]	.03 (-.01;.08)	.02 (-.02;.07)	.04 (-.002;.09)	.03 (-.02;.07)	.02 (-.02;.07)	.03 (-.01;.08)	.04 (-.01;.08)	.04 (-.01;.08)	.04 (-.01;.08)
Occupational status: Permanent contract[a]	.03 (-.01;.08)	.02 (-.02;.07)	.03 (-.01;.07)	.01 (-.03;.06)	.02 (-.03;.06)	.02 (-.03;.06)	.01 (-.03;.05)	.04 (-.01;.08)	.03 (-.01;.08)
Relationship status: Widowed[a]	.05 (-.01;.11)	-.02 (-.09;.04)	.05 (-.01;.11)	.01 (-.05;.07)	.04 (-.02;.10)	.01 (-.05;.07)	.05 (-.02;.11)	.03 (-.03;.10)	.05 (-.01;.11)
Relationship status: Divorced[a]	-.08 (-.25;.09)	-.13 (-.30;.04)	-.09 (-.26;.09)	-.13 (-.29;.04)	-.09 (-.27;.08)	-.11 (-.28;.06)	-.10 (-.27;.07)	-.10 (-.27;.08)	-.06 (-.23;.11)
Relationship status: Separated[a]	-.002 (-.11;.10)	.003 (-.10;.11)	.03 (-.08;.13)	-.03 (-.14;.07)	-.03 (-.13;.08)	-.04 (-.14;.07)	-.01 (-.11;.09)	.01 (-.10;.12)	.003 (-.10;.11)

Relationship status: Domestic	.02 (-.15;.18)	.01 (-.16;.18)	.01 (-.15;.18)	-.01 (-.18;.15)	.01 (-.16;.17)	.001 (-.16;.16)	-.01 (-.18;.15)	.003 (-.17;.17)	.01 (-.16;.17)
Relationship status: Steady relationship, living together[a]	.09 (-.03;.20)	-.001 (-.11;.11)	.07 (-.04;.18)	.06 (-.05;.17)	.06 (-.05;.17)	.06 (-.05;.17)	.09 (-.02;.20)	.06 (-.06;.17)	.10 (-.01;.21)
Relationship status: Steady relationship, living apart[a]	.10 (.04;.17)***	.04 (-.03;.10)	.11 (.04;.17)***	.09 (.02;.15)**	.10 (.04;.16)***	.07 (.01;.13)*	.10 (.03;.16)***	.10 (.03;.16)**	.11 (.04;.17)***
Relationship status: Single[a]	.03 (-.03;.10)	-.04 (-.11;.03)	.03 (-.04;.10)	.02 (-.05;.09)	.03 (-.04;.10)	.03 (-.04;.10)	.04 (-.03;.11)	.02 (-.05;.09)	.03 (-.04;.09)
Relationship status: Other[a]	.14 (-.05;.32)	.13 (-.05;.32)	.15 (-.03;.34)	.14 (-.05;.32)	.14 (-.05;.32)	.12 (-.06;.30)	.12 (-.06;.30)	.15 (-.04;.33)	.13 (-.06;.31)
People in household	.03 (.002;.05)*	.02 (-.001;.05)	.04 (.01;.06)**	.03 (.01;.05)*	.04 (.02;.07)***	.03 (.005;.05)*	.03 (.01;.06)**	.03 (.01;.06)*	.02 (-.004;.05)
People underage (<18) in household	.01 (-.01;.02)	.01 (-.004;.02)	.01 (-.01;.02)	.003 (-.01;.02)	.01 (-.01;.02)	.01 (-.01;.02)	.01 (-.01;.02)	.01 (-.01;.02)	.01 (-.01;.02)
General health status	.09 (.07;.11)***	.11 (.09;.13)***	.13 (.11;.15)***	.07 (.05;.09)***	.08 (.06;.10)***	.04 (.02;.06)***	.06 (.04;.08)***	.13 (.11;.15)***	.11 (.09;.12)***
Opinion about authorities' measures	.08 (.06;.10)***	.07 (.05;.09)***	.09 (.07;.11)***	.07 (.05;.09)***	.08 (.06;.11)***	.08 (.06;.10)***	.09 (.07;.11)***	.09 (.07;.11)***	.05 (.03;.07)***
Constant	-.68 (-.83;-.52)***	-.65 (-.80;-.49)***	-.86 (-1.02;-.71)***	-.55 (-.70;-.39)***	-.66 (-.82;-.51)***	-.57 (-.72;-.42)***	-.69 (-.84;-.54)***	-.85 (-1.01;-.69)***	-.59 (-.75;-.43)***
Adjusted R^2 increase by RF	0.05	0.03	0.01	0.06	0.04	0.08	0.07	0	0.04
Observations	15,790	15,790	15,790	15,790	15,790	15,790	15,790	15,790	15,790
R^2	.13	.12	.09	.15	.13	.17	.15	.09	.13
Adjusted R^2	.13	.12	.09	.15	.13	.16	.15	.08	.13
Residual Std. Error (df = 15751)	.93	.94	.95	.92	.94	.91	.92	.96	.93
F Statistic (df = 38; 15751)	64.15***	56.51***	43.20***	73.55***	60.75***	82.84***	75.55***	39.01***	63.14***

Note: **p*<0.01; ***p*<0.001; ****p*<0.0001

99% CI in parentheses

[a]Reference categories: Male (*Gender*), 18-30 (*Age*), Germany (*Country of residence*), €0-€4,999 (*Household income*), <12 yrs (*Education*), Not a student / working in research/education (*Occupation*), No permanent contract (*Occupational status*), Married (*Relationship status*)

Table S9: Tests of directed hypotheses H1-H9 (outcome: resilience to general stressors, RES_G). Note: a multiple regression was calculated separately for each hypothesized resilience factor.

Table S10: Tests of directed hypotheses H1-H9 (outcome: resilience to Corona crisis-specific stressors, RES_S)

	DV: RES_S (E_S; *SSM*)								
PAS	.26 (.24;.28)***								
PSS		.25 (.23;.27)***							
CSS			.14 (.12;.16)***						
OPT				.32 (.30;.34)***					
GSE					.27 (.25;.29)***				
REC						.36 (.34;.38)***			
NEU							-.32 (-.34;-.30)***		
BCS								.11 (.09;.13)***	
PAC									.24 (.22;.26)***
Gender: Female[a]	-.12 (-.17;-.08)***	-.18 (-.22;-.13)***	-.12 (-.16;-.07)***	-.12 (-.17;-.08)***	-.10 (-.14;-.06)***	-.05 (-.09;-.01)*	-.01 (-.05;.03)	-.15 (-.20;-.11)***	-.13 (-.18;-.09)***
Gender: Diverse[a]	-.26 (-.46;-.05)*	-.28 (-.49;-.08)**	-.26 (-.47;-.06)*	-.21 (-.41;-.01)*	-.18 (-.38;.03)	-.10 (-.29;.10)	-.13 (-.33;.07)	-.29 (-.50;-.08)**	-.26 (-.47;-.06)**
Age: 31-45	.10 (.04;.15)***	.14 (.09;.20)***	.10 (.04;.15)***	.08 (.02;.13)**	.06 (.01;.12)*	.07 (.02;.13)**	.06 (.005;.12)*	.10 (.05;.16)***	.08 (.03;.14)**
Age: 46-60	.20 (.13;.26)***	.26 (.19;.32)***	.19 (.13;.26)***	.14 (.08;.20)***	.15 (.09;.21)***	.13 (.07;.19)***	.10 (.04;.16)***	.22 (.15;.28)***	.18 (.12;.25)***
Age: 61+	.29 (.21;.37)***	.34 (.26;.43)***	.26 (.17;.34)***	.21 (.12;.29)***	.22 (.14;.31)***	.17 (.09;.25)***	.16 (.08;.25)***	.32 (.23;.40)***	.29 (.20;.37)***
Country of residence: Belgium[a]	-.12 (-.22;-.02)*	-.13 (-.23;-.03)*	-.12 (-.22;-.02)*	-.09 (-.18;.01)	-.09 (-.19;.01)	-.08 (-.17;.02)	-.09 (-.19;.01)	-.12 (-.22;-.01)*	-.18 (-.28;-.08)***
Country of residence: Hong Kong[a]	-.17 (-.27;-.08)***	-.12 (-.22;-.02)*	-.15 (-.25;-.05)***	-.04 (-.14;.05)	.05 (-.04;.15)	-.02 (-.11;.08)	-.07 (-.17;.02)	-.17 (-.27;-.06)***	-.23 (-.33;-.13)***
Country of residence: Hungary[a]	-.35 (-.44;-.25)***	-.38 (-.48;-.28)***	-.38 (-.48;-.28)***	-.37 (-.46;-.27)***	-.43 (-.53;-.34)***	-.32 (-.42;-.22)***	-.35 (-.45;-.25)***	-.43 (-.53;-.33)***	-.39 (-.49;-.29)***
Country of residence: Italy[a]	-.13 (-.23;-.04)**	-.06 (-.16;.04)	-.14 (-.24;-.04)**	-.10 (-.20;-.01)*	-.14 (-.23;-.04)**	-.12 (-.21;-.02)*	-.14 (-.23;-.04)**	-.15 (-.25;-.05)***	-.21 (-.31;-.11)***
Country of residence: Netherlands[a]	-.32 (-.38;-.25)***	-.33 (-.39;-.26)***	-.30 (-.37;-.24)***	-.30 (-.37;-.24)***	-.30 (-.36;-.23)***	-.26 (-.32;-.19)***	-.32 (-.38;-.25)***	-.32 (-.39;-.25)***	-.33 (-.40;-.27)***
Country of residence: Poland[a]	-.16 (-.23;-.09)***	-.13 (-.21;-.06)***	-.23 (-.30;-.15)***	-.12 (-.19;-.05)***	-.18 (-.25;-.11)***	-.11 (-.18;-.04)**	-.09 (-.16;-.02)*	-.22 (-.29;-.14)***	-.17 (-.24;-.10)***
Country of residence: Serbia[a]	.21 (.10;.33)***	.23 (.11;.35)***	.23 (.11;.34)***	.22 (.11;.34)***	.18 (.06;.29)***	.21 (.10;.32)***	.25 (.14;.36)***	.26 (.14;.38)***	.21 (.09;.33)***
Country of residence: Other[a]	-.02 (-.07;.04)	.04 (-.01;.10)	-.004 (-.06;.05)	-.003 (-.06;.05)	.005 (-.05;.06)	-.004 (-.06;.05)	.005 (-.05;.06)	.01 (-.05;.06)	-.05 (-.11;.002)
Household income: €5,000-€9,999[a]	-.003 (-.09;.09)	-.01 (-.10;.08)	-.02 (-.11;.07)	.01 (-.08;.10)	-.02 (-.11;.07)	-.01 (-.10;.08)	-.02 (-.11;.07)	-.02 (-.11;.08)	.01 (-.08;.10)
Household income: €10,000-€14,999[a]	.01 (-.08;.10)	-.02 (-.10;.07)	-.01 (-.10;.08)	-.01 (-.10;.08)	-.03 (-.12;.06)	-.01 (-.10;.07)	-.01 (-.10;.08)	-.01 (-.10;.08)	.02 (-.07;.11)
Household income: €15,000-€24,999[a]	.06 (-.02;.14)	.03 (-.05;.11)	.05 (-.04;.13)	.05 (-.03;.13)	.02 (-.06;.10)	.05 (-.03;.13)	.05 (-.03;.12)	.05 (-.04;.13)	.08 (-.005;.16)
Household income: €25,000-€49,999[a]	.09 (.01;.17)*	.05 (-.03;.12)	.08 (.0002;.16)*	.07 (-.01;.14)	.03 (-.04;.11)	.04 (-.03;.12)	.07 (-.01;.14)	.08 (.002;.16)*	.10 (.02;.18)**
Household income: €50,000-€74,999[a]	.10 (.02;.19)*	.06 (-.02;.15)	.09 (.01;.18)*	.08 (-.001;.16)	.04 (-.04;.13)	.05 (-.04;.13)	.06 (-.02;.14)	.10 (.01;.19)*	.11 (.03;.20)**
Household income: €75,000-€99,999[a]	.09 (-.001;.19)	.05 (-.05;.14)	.10 (-.002;.20)	.07 (-.03;.16)	.04 (-.06;.13)	.03 (-.06;.12)	.06 (-.04;.15)	.10 (.002;.20)*	.12 (.02;.22)*
Household income: €10,000+[a]	.11 (.02;.20)*	.07 (-.03;.16)	.12 (.02;.21)*	.09 (.003;.18)*	.04 (-.05;.13)	.03 (-.06;.12)	.06 (-.03;.15)	.12 (.02;.21)*	.13 (.04;.22)**
Education: 13-16yrs[a]	.10 (.03;.18)**	.11 (.04;.19)**	.13 (.05;.20)***	.11 (.04;.19)***	.10 (.03;.18)**	.12 (.05;.19)***	.11 (.04;.19)***	.12 (.05;.20)***	.12 (.05;.20)***
Education: 17-20yrs[a]	.14 (.07;.21)***	.15 (.07;.22)***	.18 (.10;.25)***	.15 (.08;.22)***	.13 (.06;.20)***	.15 (.08;.23)***	.15 (.08;.23)***	.16 (.09;.24)***	.16 (.09;.23)***
Education: 21+ yrs[a]	.20 (.12;.29)***	.22 (.13;.30)***	.24 (.15;.33)***	.21 (.13;.30)***	.19 (.10;.27)***	.21 (.13;.29)***	.22 (.13;.30)***	.23 (.14;.32)***	.22 (.14;.31)***
Occupation: Student / working in research/education[a]	-.01 (-.06;.03)	-.02 (-.07;.02)	-.002 (-.05;.04)	-.02 (-.06;.02)	-.03 (-.07;.02)	-.01 (-.06;.03)	-.01 (-.05;.04)	-.01 (-.05;.04)	-.01 (-.05;.04)
Occupational status: Permanent contract[a]	.10 (.06;.14)***	.09 (.05;.13)***	.09 (.05;.14)***	.08 (.04;.12)***	.08 (.04;.12)***	.08 (.04;.12)***	.07 (.03;.12)***	.10 (.06;.15)***	.10 (.06;.14)***

Relationship status: Widowed[a]	.13 (.07;.19)***	.05 (-.01;.11)	.13 (.07;.20)***	.10 (.04;.16)***	.12 (.06;.18)***	.09 (.03;.15)***	.13 (.07;.19)***	.12 (.05;.18)***	.13 (.07;.19)***
Relationship status: Divorced[a]	-.06 (-.22;.11)	-.12 (-.28;.05)	-.07 (-.24;.11)	-.11 (-.27;.06)	-.07 (-.24;.09)	-.09 (-.25;.07)	-.08 (-.24;.09)	-.08 (-.25;.09)	-.04 (-.21;.13)
Relationship status: Separated[a]	.03 (-.07;.13)	.03 (-.07;.14)	.06 (-.04;.17)	-.005 (-.10;.10)	-.0003 (-.10;.10)	-.01 (-.11;.09)	.02 (-.08;.12)	.04 (-.06;.15)	.04 (-.07;.14)
Relationship status: Domestic	.01 (-.15;.17)	.004 (-.16;.17)	.01 (-.16;.17)	-.02 (-.18;.14)	.001 (-.16;.16)	-.01 (-.17;.15)	-.02 (-.18;.14)	-.005 (-.17;.16)	-.003 (-.17;.16)
Relationship status: Steady relationship, living together[a]	.17 (.06;.28)***	.07 (-.04;.18)	.15 (.04;.27)**	.14 (.03;.25)**	.14 (.04;.25)**	.14 (.04;.25)**	.17 (.07;.28)***	.14 (.02;.25)*	.18 (.07;.29)***
Relationship status: Steady relationship, living apart[a]	.16 (.10;.22)***	.08 (.02;.15)**	.16 (.10;.23)***	.14 (.08;.20)***	.15 (.09;.22)***	.12 (.06;.19)***	.15 (.09;.21)***	.15 (.08;.21)***	.16 (.10;.23)***
Relationship status: Single[a]	.03 (-.04;.10)	-.05 (-.12;.02)	.03 (-.04;.09)	.01 (-.06;.08)	.02 (-.05;.09)	.02 (-.04;.09)	.03 (-.03;.10)	.01 (-.06;.08)	.02 (-.05;.09)
Relationship status: Other[a]	.05 (-.13;.23)	.05 (-.13;.23)	.07 (-.12;.25)	.05 (-.13;.23)	.05 (-.13;.23)	.03 (-.14;.21)	.04 (-.14;.21)	.06 (-.13;.25)	.04 (-.14;.23)
People in household	.02 (-.01;.04)	.02 (-.01;.04)	.03 (.01;.06)*	.02 (-.002;.05)	.03 (.01;.06)**	.02 (-.002;.05)	.03 (.003;.05)*	.02 (-.001;.05)	.01 (-.01;.04)
People underage (<18) in household	.01 (-.001;.03)	.02 (.004;.03)**	.02 (.002;.03)*	.01 (-.003;.02)	.01 (.001;.03)*	.01 (-.0002;.03)	.01 (.002;.03)*	.01 (.0002;.03)*	.01 (.001;.03)*
General health status	.09 (.07;.11)***	.11 (.09;.13)***	.13 (.11;.15)***	.07 (.05;.09)***	.08 (.06;.10)***	.04 (.02;.05)***	.05 (.04;.07)***	.13 (.11;.15)***	.11 (.09;.13)***
Opinion about authorities' measures	.07 (.05;.09)***	.06 (.04;.08)***	.08 (.06;.10)***	.06 (.04;.08)***	.08 (.06;.10)***	.07 (.05;.09)***	.08 (.06;.10)***	.09 (.07;.11)***	.04 (.02;.06)***
Constant	-.79 (-.94;-.64)***	-.73 (-.89;-.58)***	-.99 (-1.14;-.83)***	-.64 (-.79;-.49)***	-.76 (-.91;-.60)***	-.66 (-.81;-.51)***	-.80 (-.95;-.65)***	-.97 (-1.12;-.81)***	-.72 (-.88;-.57)***
Adjusted R^2 increase by RF	0.08	0.07	0.03	0.1	0.08	0.12	0.1	0.03	0.07
Observations	15,790	15,790	15,790	15,790	15,790	15,790	15,790	15,790	15,790
R^2	.17	.16	.12	.19	.17	.21	.19	.11	.16
Adjusted R^2	.16	.16	.12	.19	.16	.21	.19	.11	.15
Residual Std. Error (df = 15751)	.91	.92	.94	.90	.91	.89	.90	.94	.92
F Statistic (df = 38; 15751)	82.93***	77.42***	57.36***	97.26***	82.63***	110.42***	97.89***	53.51***	76.93***

Note: $^{*}p<0.01$; $^{**}p<0.001$; $^{***}p<0.0001$

99% CI in parentheses

[a]Reference categories: Male (*Gender*), 18-30 (*Age*), Germany (*Country of residence*), €0-€4,999 (*Household income*), <12 yrs (*Education*), Not a student / working in research/education (*Occupation*), No permanent contract (*Occupational status*), Married (*Relationship status*)

Table S10: Tests of directed hypotheses H1-H9 (outcome: resilience to Corona crisis-specific stressors, RES_S). Note: a multiple regression was calculated separately for each hypothesized resilience factor.

4 Incomplete data

After data cleaning (see main text), there were n=2905 valid but incomplete data sets. Of these, 2849 did not contain complete answers to the questions on stressor exposure, which were placed at the end of the questionnaire. As this precludes the calculation of the outcome variable resilience (mental health normalized to stressor exposure, see 2.3.4), the incomplete data sets were as a whole not included in the analyses. Qualitative comparison with the participants providing complete data (Table S1) indicated that the participants providing incomplete data were more likely male and younger, were more likely not to report a mental health diagnosis, to live in a household with 3-4 people and with underage people, to have fewer years of education and a lower average household income and to undergo education, while being less likely to work in education or research. They were also more likely to be Polish residents or nationals and less likely to be German residents or nationals. We suspect that younger participants may have been less patient with filling in the 127-item questionnaire.

5 References

Amstadter AB, Myers JM, Kendler KS (2014) Psychiatric resilience: longitudinal twin study. Br J Psychiatry 205:275–280.

Beierlein C, Kovaleva A, Kemper CJ, Rammstedt B (2012) ASKU - Allgemeine Selbstwirksamkeit Kurzskala. Messinstrument zur Erfassung subjektiver Kompetenzerwartungen Available at: https://www.psycharchives.org/handle/20.500.12034/431.

Bonanno GA, Mancini AD (2011) Toward a lifespan approach to resilience and potential trauma. In: Resilience and mental health: challenges across the lifespan, pp 120–134. Cambridge, UK: Cambridge University Press.

Bonanno GA, Romero SA, Klein SI (2015) The Temporal Elements of Psychological Resilience: An Integrative Framework for the Study of Individuals, Families, and Communities. Psychol Inq 26:139–169.

Carver CS (1997) You want to measure coping but your protocol's too long: consider the brief COPE. Int J Behav Med 4:92–100.

Chmitorz A, Kurth K, Mey LK, Wenzel M, Lieb K, Tüscher O, Kubiak T, Kalisch R (2020) Assessment of Microstressors in Adults: Questionnaire Development and Ecological Validation of the Mainz Inventory of Microstressors. JMIR Ment Health 7:e14566.

Dunkel D, Antretter E, Fröhlich-Walser S, Haring C (2005) [Evaluation of the short-form social support questionnaire (SOZU-K-22) in clinical and non-clinical samples]. Psychother Psychosom Med Psychol 55:266–277.

Garnefski N, Kraaij V (2006) Cognitive emotion regulation questionnaire – development of a short 18-item version (CERQ-short). Pers Indiv Diff 41:1045–1053.

Goldberg DP, Gater R, Sartorius N, Ustun TB, Piccinelli M, Gureje O, Rutter C (1997) The validity of two versions of the GHQ in the WHO study of mental illness in general health care. Psychol Med 27:191–197.

Jeronimus BF, Kotov R, Riese H, Ormel J (2016) Neuroticism's prospective association with mental disorders halves after adjustment for baseline symptoms and psychiatric history, but the adjusted association hardly decays with time: a meta-analysis on 59 longitudinal/prospective studies with 443 313 participants. Psychol Med 46:2883–2906.

Jorm AF, Patten SB, Brugha TS, Mojtabai R (2017) Has increased provision of treatment reduced the prevalence of common mental disorders? Review of the evidence from four countries. World Psychiatry 16:90–99.

Kalisch R et al. (2017) The resilience framework as a strategy to combat stress-related disorders. Nat Hum Behav 1:784.

Kalisch R et al. (2020) A generic solution for the operationalization and measurement of resilience and resilience processes in longitudinal observations: rationale and basic design of the MARP and LORA studies. psyRxiv, doi: 10.31234/osf.io/jg238.

Kalisch R, Müller MB, Tüscher O (2015) A conceptual framework for the neurobiological study of resilience. Behav Brain Sci:e92.

Kampa M, Schick A, Sebastian A, Wessa M, Tüscher O, Kalisch R, Yuen K (2020) Replication of fMRI group activations in the neuroimaging battery for the Mainz Resilience Project (MARP). Neuroimage 204:116223.

Kampa M, Schick A, Yuen K, Sebastian A, Chmitorz A, Saase V, Wessa M, Tüscher O, Kalisch R (2018) A Combined Behavioral and Neuroimaging Battery to Test Positive Appraisal Style Theory of Resilience in Longitudinal Studies. bioRxiv, doi: 10.1101/470435.

Mancini AD, Bonanno GA (2009) Predictors and Parameters of Resilience to Loss: Toward an Individual Differences Model. J Pers 77:1805–1832.

Rammstedt B, John OP (2007) Measuring personality in one minute or less: A 10-item short version of the Big Five Inventory in English and German. J Res Pers 41:203–212.

Sapienza JK, Masten AS (2011) Understanding and promoting resilience in children and youth. Curr Opin Psychiatry 24:267–273.

Schenk HM, Jeronimus BF, van der Krieke L, Bos EH, de Jonge P, Rosmalen JGM (2018) Associations of Positive Affect and Negative Affect With Allostatic Load: A Lifelines Cohort Study. Psychosom Med 80:160–166.

Smith BW, Dalen J, Wiggins K, Tooley E, Christopher P, Bernard J (2008) The brief resilience scale: assessing the ability to bounce back. Int J Behav Med 15:194–200.

van Harmelen A-L, Kievit RA, Ioannidis K, Neufeld S, Jones PB, Bullmore E, Dolan R, NSPN Consortium, Fonagy P, Goodyer I (2017) Adolescent friendships predict later resilient functioning across psychosocial domains in a healthy community cohort. Psychol Med 47:2312–2322.

Watson D, Clark LA, Tellegen A (1988) Development and validation of brief measures of positive and negative affect: the PANAS scales. J Pers Soc Psychol 54:1063–1070.
